# Top-down control of cortical gamma-band communication via pulvinar induced phase shifts in the alpha rhythm

**DOI:** 10.1371/journal.pcbi.1005519

**Published:** 2017-05-04

**Authors:** Silvan Quax, Ole Jensen, Paul Tiesinga

**Affiliations:** 1 Department of Neuroinformatics, Donders Institute, Radboud University, Nijmegen, The Netherlands; 2 School of Psychology, Centre for Human Brain Health, University of Birmingham, Birmingham, United Kingdom; University College London, UNITED KINGDOM

## Abstract

Selective routing of information between cortical areas is required in order to combine different sources of information according to cognitive demand. Recent experiments have suggested that alpha band activity originating from the pulvinar coordinates this inter-areal cortical communication. Using a computer model we investigated whether top-down induced shifts in the relative alpha phase between two cortical areas could modulate cortical communication, quantified in terms of changes in gamma band coherence between them. The network model was comprised of two uni-directionally connected neuronal populations of spiking neurons, each representing a cortical area. We find that the phase difference of the alpha oscillations modulating the two neuronal populations strongly affected the interregional gamma-band neuronal coherence. We confirmed that a higher gamma band coherence also resulted in more efficient transmission of spiking information between cortical areas, thereby confirming the value of gamma coherence as a proxy for cortical information transmission. In a model where both neuronal populations were connected bi-directionally, the relative alpha phase determined the directionality of communication between the populations. Our results show the feasibility of a physiological realistic mechanism for routing information in the brain based on coupled oscillations. Our model results in a set of testable predictions regarding phase shifts in alpha oscillations under different task demands requiring experimental quantification of neuronal oscillations in different regions in e.g. attention paradigms.

## Author summary

Cortical oscillations have been linked to the process of communication between two brain areas. Here we investigated how a third area could control communication between two other brain areas. We find that the phase of a slower alpha-band oscillation is able to influence the power of faster gamma oscillations. By changing phase differences between the slower oscillation in two areas, a third area is able to control the amount of information flow. In a network with bi-directional connections, the direction of communication is also controlled by this phase difference. Our results suggest that the pulvinar could coordinate communication between different brain areas. This area could have a central role in prioritizing the processing of sensory information that is most relevant for the task at hand.

## Introduction

The selective routing of information between neocortical areas is important for efficient task-specific communication. Since it is impossible for the brain to simultaneously process all the incoming sensory information, it is crucial to enhance the processing of the most relevant information for the task at hand while at the same time ignoring irrelevant or distracting information. This process of selective attention modulates neural activity both in early visual as well as their down-stream areas [1]. Since tasks and goals are represented in higher-order cortical areas, there is a need for a mechanism by which these higher-order areas can influence information processing in lower-order areas. We here explore a mechanism whereby the communication between two lower-order sensory areas is coordinated by a third area inducing phase shifts in slow oscillations. This coordination serves to increase or decrease the efficiency of information transfer depending on task demands. We use the coherence between fast oscillations in these two areas as a proxy for efficiency of information transfer.

Synchronization between different cortical rhythms has been suggested as a means for selective communication between cortical areas [2–4]. Experiments in monkeys have shown specifically that synchronization of the gamma rhythm between cortical areas in the occipital cortex is linked to the selective processing of information during visual attention tasks [5]. However, the mechanism by which this selective synchrony modulation of the gamma rhythm is coordinated in a top-down manner remains unknown. One proposal is that the synchronization is achieved by feedforward entrainment, in which a sending region drives the receiving region [6].

There is in addition an abundance of evidence linking the alpha rhythm to the attentional processing of information. Alpha power has specifically been shown to correlate with the level of attentional processing of information [7–10]. When a hemisphere responds to an attended stimulus, alpha power decreases in this hemisphere, lifting inhibition and increasing information processing abilities. At the same time increases in alpha power inhibit processing of information in the other hemisphere. [11–13].

The cortical alpha rhythm is well studied, but its origins remain unclear. There is strong evidence for alpha generators in the infragranular layers of the cortex [14–17]. However, experiments also show that the neocortical alpha activity is coherent with the thalamus [18,19], suggesting that thalamic activity might entrain these infragranular sources. The study by Saalmann and coworkers has linked the alpha rhythm originating from the pulvinar to attentional processing of information in the cortex [20]. The pulvinar has widespread connections to virtually every part of the visual cortex [21,22]. When two cortical areas are connected directly by cortico-cortical connections they also receive projections from overlapping populations in the pulvinar [23]. Hence, the pulvinar is an ideal candidate for coordinating the communication between cortical areas. The study by Saalmann and coworkers has shown that selective allocation of attention was associated with an increase in Granger causality (GC) from the pulvinar to the parts of the visual cortex that respond to the attended stimuli, lending further support to the idea that the pulvinar coordinates information transmission between cortical areas. Furthermore, this study showed an attention-dependent increase in gamma band as well as alpha band coherence between the relevant parts of cortical areas V4 and TEO, which was correlated with a significant increase of alpha band coherence between the pulvinar and these two cortical areas [20].

In another study, electrical stimulation of the pulvinar caused an increase in firing rate of a cortical neuron when its receptive field (RF) overlapped with the receptive field of the stimulated pulvinar neurons, while it lowered the firing rate when the receptive field of the cortical neuron and the pulvinar neurons did not overlap [24]. This indicates that pulvinar projections can enhance or suppress activity in the cortex, possibly influencing cortico-cortical communication.

The experimental observation of cross-frequency coupling between alpha phase and gamma band power supports the idea that the alpha rhythm coordinates neuronal processes at higher frequencies [16,25]. How this rhythmic modulation of gamma power influences communication remains unknown. As mentioned in the preceding text it has been suggested that increases in alpha power decrease gamma activity in a cortical area, thereby reducing its ability to transmit this information to downstream areas [8]. Another possibility is that the alpha phase modulation of gamma power leads to windows of high gamma power, which can be aligned across different areas to improve transmission. In contrast to the idea that gamma band synchronization is caused by feed-forward entrainment, this would require that top-down modulation of the alpha phase is able to influence gamma band synchronization by adjusting alpha phases in different areas.

Experiments conducted to study the role of the alpha phase in cortical communication have focused mainly on determining whether the perception of stimuli depends on the alpha phase at which they were presented [26,27]. Recent experiments show that the brain can either actively adjust its alpha phase during an attentional distractor task [28,29] or modulate the power [30]. These studies have focused on alpha phase adjustments in tasks with temporal expectations about the onset of stimuli. Although this is often behaviorally relevant, the brain also needs a mechanism to enhance processing of uncued stimuli. A possible mechanism would be to align the alpha phase between different cortical areas rather than adjusting the alpha phase with respect to stimulus onset, thereby making the enhancing effect independent of stimulus onset.

Here we investigate by means of a model network comprised of spiking neurons whether the pulvinar could coordinate communication in the gamma band between two cortical areas by aligning the relative alpha phase between these cortical areas. We find that the phase difference between the alpha rhythms of the two populations influences coherence in the gamma band, as well as the amount of stimulus information sent from one area to another. In bi-directional networks we were also able to control the direction of communication by adjusting the alpha phase difference. These results account for a broad set of experimental studies of the role of alpha and gamma oscillations in attentional processing of information.

## Results

### Gamma power coupled to alpha phase

**Fig 1:**
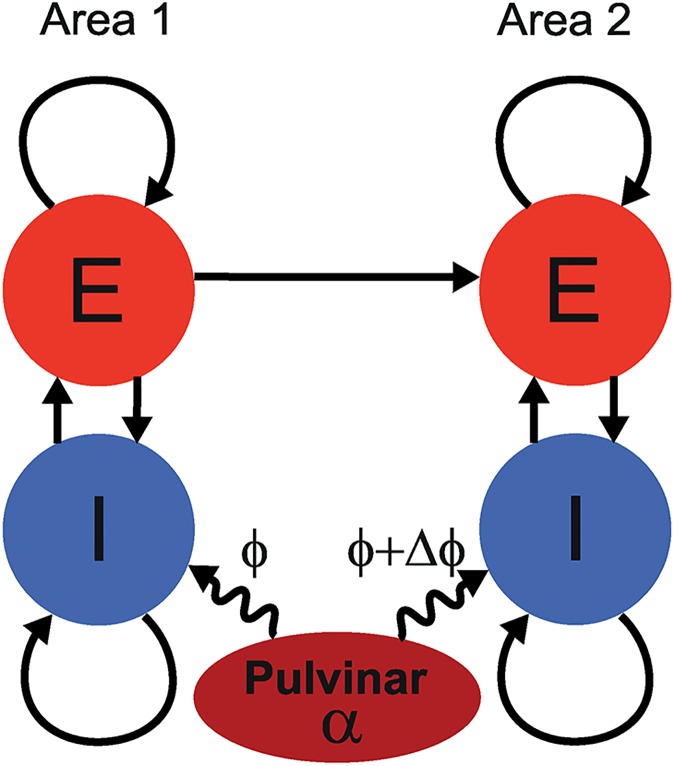
Schematic of model used in the simulations. Two neocortical areas were uni-directionally connected via a feedforward connection. Both areas consisted of excitatory (RS) neurons and inhibitory (FS, LTS) neurons. Reciprocal connections existed between the excitatory and inhibitory populations within one area. Neurons of each cell type were also recurrently connected within each area. The input to the FS neurons was modulated by an alpha-band oscillatory drive from the pulvinar. The pulvinar neurons were not explicitly modeled. The effect of varying the relative alpha phase Δϕ on communication between the neocortical areas was tested.

Consider a network of two connected neocortical areas coordinated by the pulvinar (Fig 1). To study communication between the neocortical areas we modeled each population as a local network of interconnected regular spiking excitatory (E) and inhibitory (I) neurons. The inhibitory populations consisted of a combination of fast spiking (FS) and low-threshold spiking (LTS) interneurons, all modeled using the Izhikevich model [31] with the appropriate parameter setting (see METHODS).

**Fig 2:**
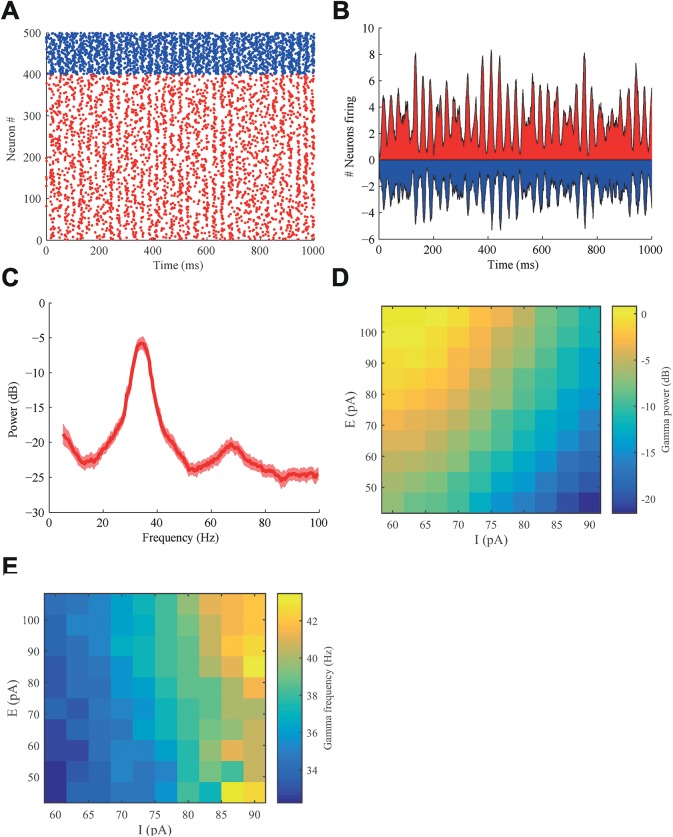
Gamma oscillations emerged spontaneously from the reciprocal interaction between E cells and I cells. (A) Rastergram of excitatory (red) and inhibitory (blue) neurons in one population during an interval of 1000 ms. Gamma oscillations are visible as vertical alignment of spike times. (B) The spike time histogram (STH) reveals the gamma oscillations (bin width Δt = 1 ms) in both excitatory and inhibitory cells. (C) The power spectral density of the E-population STH, showing gamma oscillations between 30 and 50 Hz. Data was averaged over 10 trials, the shaded areas represent the standard error of the mean (SEM). (D) Increasing the input current to excitatory neurons increases gamma power, while increasing input current to the inhibitory neurons decreases gamma power. The current to excitatory cells is varied along the y-direction, whereas that to the inhibitory cells is varied along the x-axis, the resulting gamma power is color-coded according to the color bar shown on the right of the panel. (E) The frequency of the gamma oscillation increases with increasing input to the excitatory as well as the inhibitory neurons.

Model parameters were adjusted such that the network produced biologically realistic firing rates, specifically 5–10 Hz for E neurons and 25–35 Hz for the two types of I neurons (see METHODS). Gamma oscillations emerged spontaneously through a pyramidal-interneuron gamma (PING) mechanism ([32]), in which excitatory input from the pyramidal neurons activated the interneurons. In return they inhibited the network for a period determined by the time scale of fast GABAergic feedback. This resulted in synchronous population activity that oscillated with a frequency in the gamma band (Fig 2A). The oscillations were not regular: the peaks in the spike time histogram (STH) as well as the period of the gamma oscillations fluctuated (Fig 2B). The majority of the analyses reported in this paper are performed on the STH signal of the E population. This is because the currents entering and leaving neurons from the E population during synaptic inputs and spiking are thought to contribute most to the extracellular local field potential measured in electrophysiological studies due to their large dipole fields [33].

A clear peak in the power spectrum is observed in the gamma band (Fig 2C). This gamma band is fairly wide reflecting the frequency fluctuations across time. The mean gamma power and frequency strongly depended on the mean input strength to the E and the I neurons (Fig 2D and 2E). The mean gamma power was determined as the average power over a symmetric 20 Hz-wide frequency band centered around the peak frequency, which was separately determined for each individual parameter setting. Increasing input to the E population resulted in an increase in gamma power, while stronger input to the I population reduced gamma power (Fig 2D). The peak frequency of the gamma band increased with stronger input to the E cells as well as the I cells (Fig 2E). These simulations show that we can modulate the power and frequency of gamma oscillations independently by setting the level of depolarization of the inhibitory and excitatory neurons to their appropriate value.

To study the coupling between the alpha rhythm and the emergent gamma oscillations we applied a modulatory input of alpha frequency (10 Hz) with an amplitude of 23 pA to the I population as described in the METHODS section. There was no alpha frequency input current to the excitatory neurons, hence the effect of the modulation on the E population was assumed to be indirect and mediated by the projection of the I population to the E population. A modulatory input to the cells in the alpha frequency band can be interpreted as a slow variation of the input currents, hence appealing to the results shown in Fig 2, we can expect the gamma power and frequency to vary with the alpha phase.

**Fig 3:**
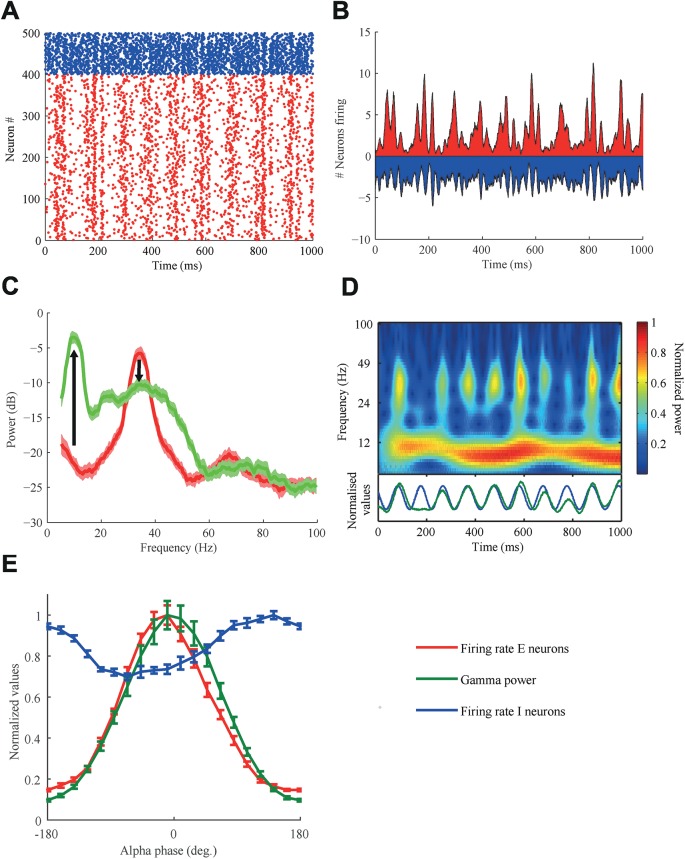
The alpha drive from the pulvinar modulates gamma power in a phasic manner. (A,B) The alpha modulation from the pulvinar drive to the I-cells is clearly visible in both (A) the rastergram and (B) the STH. (C) The power spectral density of the E-cell STH had peaks both in the alpha and gamma band. Data was averaged over 10 trials, the shaded areas represent the standard error of the mean (SEM). (D) The gamma power was modulated by the phase of the alpha oscillation. Top: The spectrogram obtained via a wavelet analysis. Bottom: The gamma power (20–50 Hz; green line) is locked to the phase of the modulating alpha oscillation (blue line). (E) The gamma power (green line) follows the peak activity of excitatory neurons (red line), whereas the peak for the inhibitory neuronal firing (blue line) is approximately out of phase with gamma power. Data was averaged over 10 trials, the error bars represent the standard error of the mean (SEM).

Under these circumstances, the STH of the E population now displayed oscillations in the gamma as well as in the alpha band (Fig 3A and 3B). These observations are also reflected in the power spectral density, which now has peaks in these two frequency bands (Fig 3C). The introduction of the alpha rhythm not only decreased the gamma power, in accordance to experimental studies [34], but also broadened the peak, reflecting the variation of gamma oscillation frequency with alpha phase. We applied a wavelet analysis to determine how the power of the gamma rhythm was coupled to the phase of the alpha rhythm. The alpha band activity was visible as a red-yellow horizontal band in the spectrogram centered around 10 Hz with only modest variations in power and frequency (Fig 3D). The gamma power was modulated by alpha phase, which was reflected in a periodic sequence of transient yellow-green blobs (Fig 3D, below). These blobs were locked to the peaks of the alpha cycle in the E population and the troughs in the I population (Fig 3E). The phase of the alpha oscillations in the E and the I populations are approximately 180° apart, which indicates that high activity of the I population inhibits the E population. The inhibitory population thus serves to gate the activity of the excitatory neurons. If the drive from the pulvinar to the cortex projects predominantly to the inhibitory cells, this predicts that when excitatory neurons in the pulvinar fire, the excitatory neurons in the cortex are inhibited. This would mean that the alpha rhythm as measured in terms of excitatory neurons in cortex is fully out of phase relative to the activity in excitatory cells of the thalamus, given that axonal delays from pulvinar to cortex are significantly shorter than an alpha cycle.

### Relative alpha phase modulates gamma coherence

In order to quantify how alpha phase modulates communication we studied uni-directionally connected networks of two neuronal populations, each representing a cortical area as described in the METHODS section (Fig 1). We used gamma band coherence between these populations as index for the level of information transmission, communication for short. We varied both the relative alpha phase between the populations as well as alpha power. The following effects of alpha phase difference were obtained using a intermediate value of the alpha amplitude of 23 pA.

**Fig 4:**
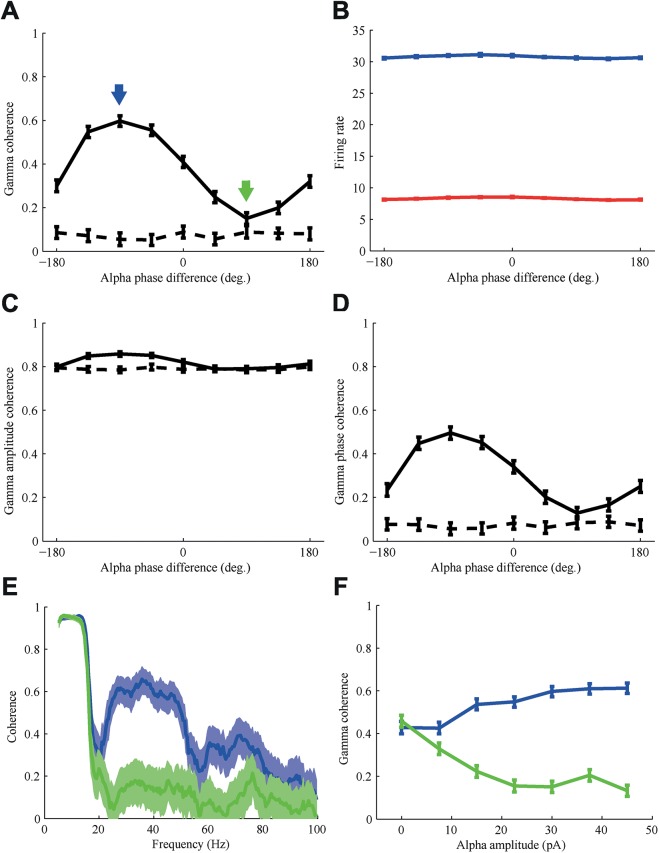
Gamma coherence is modulated by the phase difference between the alpha oscillations from the pulvinar that each area receives. For all panels data was averaged over 10 trials, the error bars or shaded areas represent the standard error of the mean (SEM). (A) Gamma coherence between area 1 and 2 depends strongly on the alpha phase difference. The blue arrow indicates the optimal and the green arrow indicates the least optimal alpha phase difference. (B) The firing rate of excitatory (red) and inhibitory (blue) neurons of the second area did not vary strongly with alpha phase difference. (C,D) The overall coherence can be resolved in a contribution due to a correlation in amplitude and a phase coherence, which are shown panel C and D, respectively. The amplitude coherence varied much less with alpha phase difference than the phase coherence. The dashed line in A,C, and D represent the bias in the coherence, which was determined by calculating the coherence between randomly shuffled trials. The amplitude coherence is more strongly biased than the phase coherence, due to the similar alpha modulation in both areas. (E) When comparing the coherence spectrum for the optimal alpha phase (-90°, blue) with the least optimal alpha phase (90°, green), the difference in coherence occurs only in to the gamma band, the peak of the coherence in the alpha band is unaffected. (F) The strength of the modulation with alpha phase increases with the amplitude of the alpha modulation, primarily by reducing the coherence at the least optimal alpha phase.

The relative alpha phase difference had a strong effect on the coherence in the gamma band (Fig 4A). The optimal alpha phase difference (Δφ^+^) for communication, i.e. the one resulting in the highest gamma coherence was approximately -90°, while the least optimal alpha phase difference (Δφ^-^), was approximately 90°. Maximal difference in gamma coherence between Δφ^+^ and Δφ^-^ was obtained when the alpha oscillation was strong enough to reduce gamma power in the sending network to zero at the peak of the alpha phase (i.e. when inhibitory activity was maximal). The alpha phase-difference leading to maximal gamma coherence corresponds to the state when area 1 leads area 2 by approximately 25 ms, or 1 gamma cycle. The most optimal and least optimal alpha phase for communication differ 180°. The overall firing rates of the excitatory and inhibitory populations in area 1 and 2 did not vary by more than 2% with alpha phase difference and can thus not be solely responsible for the much larger modulation of the gamma coherence (Fig 4B).

The coherence measure receives contributions from two different properties of a signal, an amplitude contribution, increasing when fluctuations across trials in amplitude between the two areas are similar, and a phase contribution, increasing when phases differences vary less across trials [35]. We were interested in determining on which component of the coherence the alpha modulation had the biggest effect. Fig 4C and 4D show the amplitude coherence and phase coherence, respectively. The amplitude coherence is very high, and strongly biased, as expected because the amplitudes in each area fluctuate similarly due to the common alpha modulation, even when compared across shuffled trials. However, the level of amplitude coherence does not vary strongly with alpha phase; rather the biggest effect is found for the gamma phase coherence. This indicates that the phases of the gamma oscillations synchronize better across areas when there is an optimal alpha phase difference. In Fig 4E the coherence spectrum for Δφ^+^ (blue) and Δφ^-^ (green) is shown. The coherence in the alpha band is close to unity because of the synchronized alpha input and differs little between most and least optimal phase. There is however a clear difference in coherence around the gamma frequencies (30–50 Hz) as well as a smaller harmonic effect around 70 Hz. These frequency bands match the peak locations in the power spectrum shown in Fig 3C. These results were obtained by modulating the input to the inhibitory neurons with an alpha oscillation. Similar results were obtained by modulating the input to the excitatory neurons (S1 Fig), except that the alpha phases were shifted by approximately 180 degrees with respect to the thalamic rhythms.

Next we modulated the alpha amplitude over a biologically relevant range to determine how this influenced the advantage of the optimal phase difference relative to least optimal phase difference. The rastergrams of neuronal firing under the lowest, middle and highest alpha amplitude can be found in S2 Fig. The modulation depth of the gamma coherence with phase difference increased with higher alpha amplitude (Fig 4F). Higher alpha amplitudes increased gamma coherence for Δφ^+^, and decreased it for Δφ^-^. The degree with which the alpha phase difference can increase gamma coherence had a sigmoidal dependence on alpha power. For lower values of the alpha amplitude no difference in gamma coherence existed, with further increases in amplitude the difference in gamma coherence increased strongly. The value of gamma coherence at the optimal alpha phase saturates with alpha power, hence further increases in alpha power do not increase maximum gamma coherence. The previous and following analyses were all performed for an intermediate level of alpha modulation amplitude equal to 23 pA (see S2B Fig for rastergram), such that there was realistic gamma activity at all alpha phases.

We implemented the alpha modulation as a sin^2^ function (while dividing the frequency by 2 such that there was still an effective frequency of 10 Hz), which has a nonzero average, so that an increased modulation depth also increased the average input to the inhibitory neurons in cortex. We considered this the more biologically plausible setting [25,36]: as stronger alpha modulation of the cortex would entail more incoming spikes from the pulvinar. In order to test whether the modulation of gamma coherence with increasing alpha amplitude was not purely due to the increases or decreases of the firing rate, we did a control simulation with the alpha modulation implemented as a sine function, which has a zero mean. Cortical firing rates for this parameter setting did not vary with increasing alpha amplitude, however the modulation of gamma coherence nevertheless remained (S3 Fig).

In conclusion, gamma coherence, used here as an index for cortical communication, can be reliably modulated by the relative alpha phase between two cortical areas.

### Coupling of spiking activity to gamma rhythms

From our previous results it is clear that alpha modulation of cortical areas influences the gamma rhythm in such a way that gamma coherence is increased or decreased. Especially the phase synchronization of the gamma rhythms in area 1 and area 2 are strongly influenced (Fig 4E). To see what the effect of this increased synchronization is on the coordination of the spiking activity of individual neurons we compared the two extreme situations of the best alpha phase difference (Δφ^+^) and the worst alpha phase difference (Δφ^-^). A wavelet analysis was used to determine the phases of the gamma rhythms in area 1 and area 2 (see METHODS).

**Fig 5:**
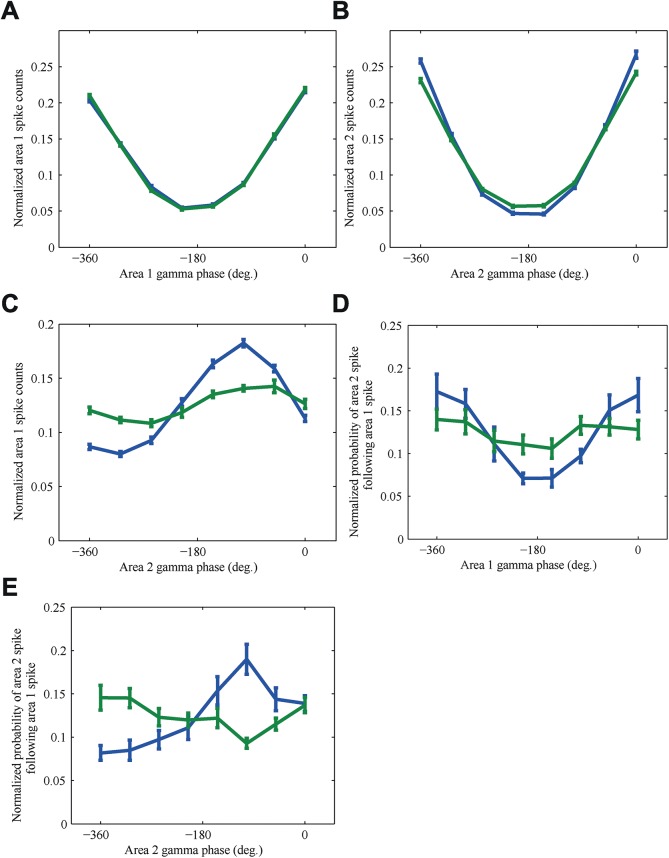
Spiking activity of individual neurons is coupled to local gamma rhythms. The conditions with the best alpha phase difference (blue) and the worst alpha phase difference (green) are compared. (A) Normalized spike counts of area 1 aligned well to the local gamma rhythm in area 1. Here, and in the two following panels, we plot the spike probability as a function of a phase normalized to 1. (B) Normalized spike counts of area 2 also aligned well to the local gamma rhythm in area 2. (C) Normalized spike counts of area 1 aligned to the non-local gamma rhythm from area 2 at a phase of approximately 90°. (D) The probability of an area 2 spike following an area 1 spike is modulated by the area 1 gamma phase. It peaks for the area 1 gamma phase at which most area 1 spikes occur. (E) Probability of an area 2 spike following an area 1 spike is modulated by the area 2 gamma phase but it peaks at a different area 2 gamma phase, namely the one where most area 1 spikes arrive rather than the phase for which most area 2 spikes occur. Data was averaged over 10 trials, the error bars represent the standard error of the mean (SEM).

The spiking activity in area 1 and area 2 were both strongly modulated by their local gamma rhythms (Fig 5A and 5B). Modulation of the spiking activity in the first area was almost the same for both conditions (Δφ^+^ vs. Δφ^-^, blue vs. green respectively), whereas in the second area the spiking activity was slightly stronger modulated for Δφ^+^ (Fig 5B). A larger difference is found when we considered how the spiking activity of area 1 aligned to the gamma rhythm in area 2 (Fig 5C). For Δφ^+^ there is a much stronger locking of area 1 spiking activity to the area 2 gamma rhythm than for Δφ^-^. Note that in both conditions the phase difference between area 1 spiking activity and the gamma rhythm of area 2 is about 90°, so even the condition with low coherence between the rhythms there is a certain amount of phase locking between spikes and the rhythms.

To see how the gamma rhythm influences the transfer of individual spikes in both conditions we calculated the probability that a spike in a neuron in area 1 is followed by a spike in a neuron in area 2 within a window of 1 to 4 ms, which matches the delays due to synaptic activation in our model (we did not include the effects of axonal delays). Our neurons never fired more than once in a 4 ms period, hence we could represent each neuron-neuron pair as a binary value. The number of spikes in area 2 was normalised over the different gamma phase bins in area 1 by randomly removing spikes from bins until each bin contained the same number of spikes. This was necessary to ensure that an increased probability is not just the result of there being more spikes in a certain gamma phase bin. We observed a strong alignment of the spike emission probability in area 2 to the gamma rhythm of area 1 for the Δφ^+^ condition, and a somewhat weaker alignment for the Δφ^-^ condition (Fig 5D). Hence, gamma phases of area 1 corresponding to a high spiking activity also lead to a higher probability that a spike in area 1 was followed by a spike in area 2.

When we quantified the probability that a spike in area 1 is followed by a spike in area 2 in terms of alignment to the gamma rhythm in area 2, we found a different effect (Fig 5E). There is still alignment for the optimal phase difference, but the gamma phases in area 2 corresponding to high spiking activity do not lead to a higher probability that a spike in area 1 is followed by a spike in area 2. Instead, for the best alpha phase difference the probability modulation agrees well with the results from Fig 5C: Gamma phases of area 2 for which more area 1 spikes occur, lead to a higher probability that a spike in area 1 is followed by a spike in area 2.

Together, these results suggest that the gamma rhythm in area 1 is responsible for increasing the effectiveness of spike transmission. Concentrating area 1 spiking activity in certain gamma phases increases area 1’s impact on area 2. The strength of this effect is modulated by the alpha phase difference between the two areas. The best alpha phase difference, for which we also found high gamma coherence, leads to a stronger modulation of the effectiveness of spike transmission by the gamma rhythm in area 1.

### Stimulus processing is affected by alpha phase

**Fig 6:**
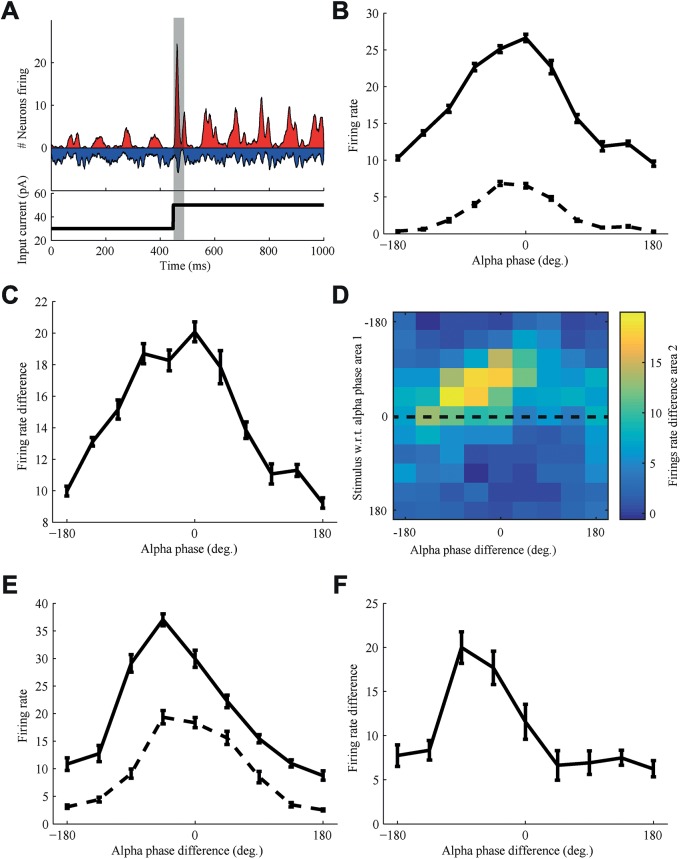
Response to a stimulus response was strongly affected by alpha phase difference. (A) Upon stimulus presentation the E and I population activity in area 1 increased strongly, yielding both a transient increase due to and immediately following the stimulus onset (highlighted by the gray bar) as well as a higher sustained rate during the period that the stimulus was presented. The bottom panel represents the stimulus time course. (B) The response of the network (firing rate during 30 ms after stimulus onset, represented by the gray bar in panel A) to the stimulus depends on at what alpha phase the stimulus onset occurs. A strong increase in response is found when the stimulus is active (solid line) compared to when there is no stimulus (dashed line). (C) The increase in firing rate of area 1 from baseline in response to the stimulus was twice as high for an optimal alpha phase compared to that for the least optimal phase. (D) The response of the second area depends both on the alpha phase of area 1 at stimulus onset (y-axis) as well as on the alpha phase difference between area 1 and area 2 (x-axis). (E) A cross section of the response surface in panel D taken at the optimal alpha phase of area 1 (0°, dashed line in panel D) highlights the effect of relative phase. Although both the baseline response (dashed line) as well as the stimulus response (solid line) are modulated by the alpha phase difference, the modulation of the latter is much stronger. (F) The difference between stimulus and baseline response is modulated by a factor of about two by the alpha phase difference between area 1 and area 2. Hence, the two phase factors have an approximately equal effect, in essence acting as a gate, which is only open if both are optimal. Data was averaged over 10 trials, the error bars represent the standard error of the mean (SEM).

An important feature of the process of selective attention is the ability to bias the processing of certain incoming stimuli over others, ensuring that the relevant stimuli have a bigger impact on downstream areas. We were interested whether the proposed mechanism of shifting alpha phases could also be used to prepare a transmission channel before the onset of a stimulus, in order to bias the processing of one stimulus over another by attention. We investigated whether shifting alpha phases can not only enhance gamma coherence and modulate spike effectiveness over a longer period of time as shown in the previous sections, but can also have an effect on short transient effects like the onset of a stimulus. Inspired by work that showed that the timing of a stimulus with respect to an ongoing cortical oscillation modulates stimulus processing [26,27], we wondered whether similar effects could be found in our network and especially whether these effects also show up when a stimulus is communicated from one cortical area to another. To investigate this we needed to present a stimulus to the model network. We modeled the incoming stimulus as an increase in excitatory input to the first neuronal population. This increase in excitatory input caused our network to go from a state with low amplitude gamma oscillations driven by an interneuron gamma (ING) mechanism, which mainly relies only on interneuron activity for the generation of gamma oscillations, to high amplitude gamma oscillations driven by a PING mechanism (Fig 6A). There was a strong initial non-linear effect on the overall firing rate of the population, after which the activity of the network returned to a state similar to the previous sections. We investigated the transient effect the stimulus had shortly (30 ms) after stimulus onset on the overall firing rate of the neuronal populations.

We quantified the response of the first population to the stimulus as the increase in firing rate during 30 ms after stimulus onset, for different moments of stimulation with respect to the phase of the alpha rhythm (Fig 6B; solid line). Since the alpha phase also modulates the firing rate without stimulation, we also quantified this baseline (Fig 6B; dashed line) and took the difference as the effective response gain caused by the stimulus (Fig 6C).

A two-times-as-strong response was found on optimal alpha phases where inhibition due to the alpha modulation was weak, compared to least optimal alpha phases where inhibition was strong. This suggests that the brain could enhance or suppress the processing of a stimulus by shifting its alpha phase, but only if it can anticipate with sufficient temporal accuracy when a stimulus would occur. Although, as mentioned in the introduction, some evidence exists for alpha phase adjustments in anticipation of a stimulus [28], the brain is also able to enhance or suppress the processing of unexpected stimuli. We hypothesize this might happen by setting up dynamical networks, or transmission channels, across which the signals are preferentially processed. To quantify this we considered the effect of the relative phase difference between two populations on the transmission of the stimulus-induced increases in firing rate of the presynaptic population.

As in Fig 1 the first population was connected to the second. This allowed us to quantify communication as the increase in firing rate in the second population, transmitted by the first population in response to a stimulus. The average firing rate over the duration of strongest response was used, which was during 30 ms after stimulus onset. The response of the second population depended both on where in the alpha phase of the first population the stimulus was presented, as well as on the relative alpha phase difference between area 1 and area 2 (Fig 6D). For the alpha phase giving the optimal response in area 1 the effect of the alpha phase difference with area 2 shows the same pattern as the gamma coherence (Fig 6E and 6F). Averaged over all stimulus onsets at the different alpha phases, the response in area 2 shows the same effect (S4A Fig). Having the optimal alpha phase difference between both populations can lead to a twice-as-strong response in the second population when compared to having the least optimal alpha phase difference.

So not only the alpha phase with respect to the incoming stimulus matters, but also the relative alpha phase between communicating areas. This shows that shifting the relative alpha phase can bias the processing of a stimulus by increasing its impact on a downstream area. Moreover a stimulus is most effectively transmitted by having the optimal alpha phase difference between successive cortical areas, while having the wrong alpha phase difference can dampen the effect a stimulus response has on downstream areas.

**Fig 7:**
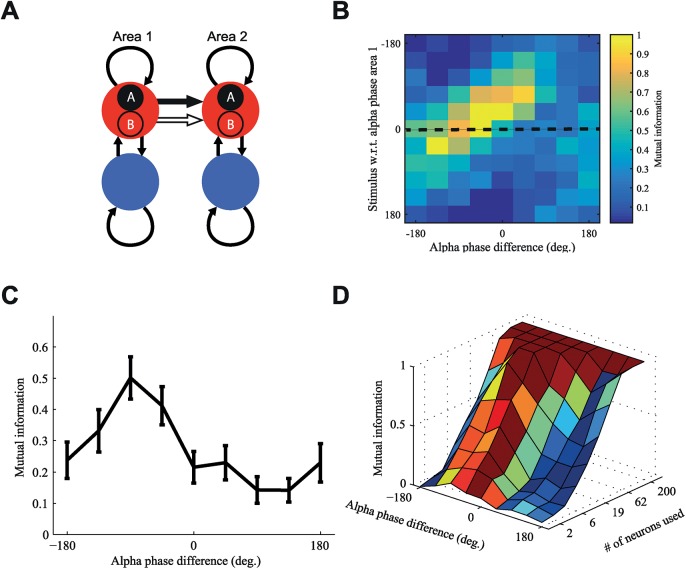
Stimulus discrimination in the receiving cortical area is influenced by alpha phase difference with the sending cortical area. (A) The excitatory populations of both areas were divided in 2 subpopulations that were individually stimulated. Each subpopulation in area 1 was selectively connected to the corresponding one in area 2. (B) The degree to which information send from the first area that could be retrieved from the response of the second area depended both on alpha phase of area 1 at stimulus onset (y-axis) as well as on the alpha phase difference between area 1 and area 2 (x-axis), in a similar fashion as the stimulus response characterized in Fig 6D. (C) A cross section at the optimal alpha phase of area 1 (0°, dashed line in panel 7D) highlights the effect of relative phase. The information transfer from area 1 to area 2 attained peak values at the same alpha phase difference where stimulus response of area 2 was highest. (D) When less neurons in area 2 were available for decoding, the performance decreased. For 200 neurons decoding performance is always 100% and the alpha phase difference is not relevant. However, for more challenging tasks for which there are fewer neurons available for decoding the optimal alpha phase difference is essential for adequate decoding. Data was averaged over 10 trials, the error bars represent the standard error of the mean (SEM).

A question that remains is whether this effect of the relative alpha phase on the impact of stimuli enhances the information that is sent from one area to another. Discriminating between different stimuli is a task that human and animal subjects can perform and that often relies on the activity of cortical areas [37] and we used this task to quantify information transfer from area 1 to area 2. We quantified the performance of the two cortical areas in discriminating two different stimuli entering at the optimal alpha phase of area 1, but arriving at different alpha phases in area 2 due to (top-down) modulation of the alpha phase difference. Each stimulus was implemented as an increase in input to a different subpopulation of the first cortical area. One can think of this as each population having slightly different stimulus preferences. These two subpopulations in area 1 each projected selectively to the two corresponding subpopulations in the second cortical area, such that there was no overlap in the feedforward projections (Fig 7A).

To quantify the discriminating abilities of the second area, we trained a support vector machine (SVM) and used this to decode which stimulus was presented to the first area. The SVM was trained on the average firing rate of the neurons in each subpopulation of the second area during 30 ms after stimulus onset, where the strongest stimulus response was observed. Classification performance depended strongly on alpha phase difference (S5A Fig). The joint probability of the actually presented stimulus and its SVM classification was used to calculate the mutual information as described in the METHODS section. Fig 7B shows how the mutual information between area 1 and area 2 depends on the relative alpha phase difference, as well as on the alpha phase of area 1 at stimulus onset. When we take the optimal alpha phase of area 1 (Fig 6C), and change the alpha phase difference between both areas we see a strong modulation of the mutual information sent from area 1 to area 2 (Fig 7C), similar to the effect of the alpha phase difference on coherence (Fig 4A) and stimulus response (Fig 6F). Averaged over all possible stimulus onsets in the alpha phase of area 1 we find the same effect (S5B Fig). The relative alpha phase thus not only increases the impact of a stimulus presented to area 1 on the response in area 2, but also increases the ability of the second area to effectively discriminate different stimuli based on inputs it received from the first area. Furthermore, when we modulate the difficulty of the discrimination task by reducing the number of neurons that can be used for decoding, we see that this dependence on alpha phase difference is only present for difficult tasks involving a smaller number of neurons (Fig 7D). When discriminating stimuli are easy the alpha phase difference doesn't matter as decoding is always performed with 100% accuracy. This supports the idea that this alpha phase dependent mechanism would only be useful for top-down processes that need to increase the performance of processing sensory information in demanding situations with limited neural resources.

### Alpha phase determines directionality of communication

The preceding results were obtained with an unidirectional connection between area 1 and area 2 whose effectiveness could be manipulated through alpha phase shifting. Brain areas are often reciprocally coupled, which would need to be modeled by bidirectional connections. To study how feedback connections influence communication we incorporated feedback connections in our model (Fig 7A). This means we cannot quantify communication solely in terms of coherence, but we also need to assess the direction of communication through analyses utilizing Granger causality.

**Fig 8:**
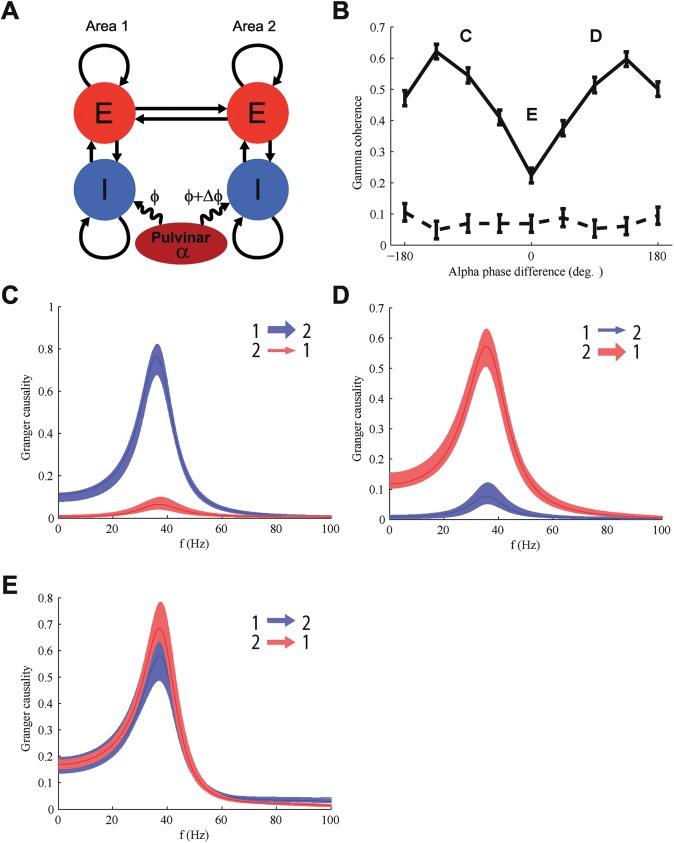
In bidirectional networks the alpha phase difference determines the direction of communication. (A) Schematic of the model with bidirectional connections between both areas. (B) With bidirectional connectivity the gamma coherence has peaks at two distinct alpha phase differences (solid line; baseline is represented by dotted line). (C) For an alpha phase difference of -90° Granger causality from area 1 to area 2 (red) is much stronger. (D) For an alpha phase difference of 90° the granger causality from area 2 to area 1 (blue) dominates. (E) At zero alpha phase difference Granger causality is equal in both directions (red, from area 1 to area 2, blue from area 2 to area 1) and shows a clear peak in the gamma band. Data was averaged over 10 trials, shaded areas represent the standard error of the mean (SEM).

In the bidirectional model simulations we varied the relative alpha phase difference between both populations. The gamma coherence showed two peaks as a function of phase difference (Fig 8B). We hypothesized that each peak corresponded to a different direction of communication.

This hypothesis was tested using the conditional Granger causality, which was conditioned on the common alpha input that both areas received (see METHODS). The relative alpha phase indeed determined the directionality of communication between the two populations (Fig 8). For negative alpha phase differences (Fig 8C) the directionality was from area 1 to area 2, while for positive phase differences (Fig 8D) the directionality was from area 2 to area 1. Causal communication was mainly in the gamma band. Without aforementioned conditioning on the common alpha input, causality in the alpha band dominated. For an alpha phase difference of 0 degrees, the causality was similar in both directions (Fig 8E) though closer inspection revealed a constantly switching directionality (S6 Fig), leading on average to lower gamma coherence (Fig 8A). The fluctuations in Granger causality occurred on timescales of between 100 and 400 ms and were probably caused by small variations in power and phase of the gamma oscillations.

## Discussion

### Summary of results

This study assessed how communication between two cortical areas, indexed by gamma coherence, can be influenced by modulation of the phase of alpha oscillations generated in the thalamus. Our results show that the phase difference between alpha rhythms in each area was a powerful modulator of the effectiveness and direction of cortical communication. Increases in alpha power can either improve or impair cortical communication, depending on the phase difference between the alpha generators. A key component of the underlying mechanism was the coupling between alpha phase and gamma power, which is often reported in experiments [25] (Fig 3D and 3E). Cortical communication was quantified in several ways to overcome the possible limitations that each individual measure might have. All measures demonstrated that communication was modulated with respect to the alpha phase difference between two areas.

The variation in gamma band coherence was primarily caused by modulation of the alpha phase difference (Fig 4A). Furthermore, we found that the alpha phase difference influences how well the arrival of individual spikes in the transmitting area is phase locked to the gamma rhythm of the receiving area (Fig 5C), thereby increasing the probability that a spike in the transmitting area evokes a spike in the receiving area.

Our simulations further show that the alpha phase difference between two areas can bias the transfer of an incoming stimulus. The response in the receiving area caused by the response to the stimulus in the transmitting area was strongly modulated by the alpha phase difference (Fig 6E and 6F). To investigate whether an enhanced impact between the two areas could be utilized to increase bandwidth for stimulus information transfer, we quantified performance on a stimulus discrimination task using mutual information.

These results also showed a strong modulation of the information transferred from the transmitting to the receiving area as a function of the alpha phase difference (Fig 7C). In a network with bidirectional connections between two cortical areas we were able to control the direction of communication by shifting the relative alpha phase. Granger causality analysis showed that gamma band communication was strong from area 1 to area 2 and weak in the reverse direction, when area 1 was leading in alpha phase (Fig 8C). The direction of gamma band GC was reversed when area 2 was leading in alpha phase instead (Fig 8D). This suggests that our observation on the link between information processing and alpha phase difference for unidirectional projections extends to bidirectional connections.

### Alpha phase to gamma power coupling

Coupling in the alpha band between thalamus and neocortical regions is well-established by animal recordings [18,19,38]. A number of recent studies report evidence for alpha-gamma cross-frequency coupling in human subjects that is consistent with the proposed model. Roux and coworkers used a beam-forming approach to show that the phase of alpha oscillations in virtual sensors located near the thalamus correlates with the amplitude of gamma oscillations in the early visual cortex [39]. A transfer entropy analysis of these data further showed that alpha phase affects gamma amplitude with a delay of about 16 ms. This is similar to physiological estimates of the transmission delay between the regions and follows from our model without explicitly building this in. Malekmohammadi and coworkers recorded simultaneously from cortex using an ECoG grid and from thalamus using depth electrodes. Their measurements show a coupling between the theta phase of oscillatory activity in the thalamus and the amplitude of beta band oscillations in cortex [40] (see also [41]). Taken together, these studies demonstrate a coupling between slow-frequency oscillations in thalamus to gamma band activity in cortex in human subjects, thereby indicating that the model studied here may be more broadly applicable to the human visual system as well.

### Alpha phase could modulate perception

The functional relevance of the phase of a low frequency oscillation (alpha or theta band) can be assessed by investigating how the detection probability of stimuli depends on the oscillatory phase at which they are presented. When visual stimuli were presented at a contrast close to detection threshold, the alpha phase for hits was different compared to that of the misses [42]. This was only true for phases calculated relative to oscillations in the theta and alpha band. Another experiment has demonstrated that not only is the detection probability modulated by alpha and theta oscillations but also that there is a phase difference in the alpha and theta frequency band between attended and unattended objects [43]. When the cyclical variation of detection probability is interpreted as being due to an oscillatory variation of neural excitability, it implies that there is a phase difference between the respective retinotopic areas responsible for processing the respective attended and unattended stimuli. This is consistent with the phase shifting between cortical areas hypothesized in the model.

### Different roles for the pulvinar in modulating attention effects

Recent experiments indicate that the thalamus, specifically the pulvinar, may be a major player in the coordination of information transmission by coordinating the synchronization between cortical areas. The simulation results reported here are relevant in relation to two recent experiments by Saalmann et al. [20] and Zhou et al. [44].

In Saalmann et al (experiment 1), monkeys are first cued to the relevant spatial location for the task, after which the stimulus is presented [20]. Upon stimulus presentation they have to respond according to the stimulus presented at the target location by either immediately releasing a lever or holding on and releasing it at a later time. Signals are recorded from the ventral pulvinar, V4 and area TEO during this task, and the analysis is conducted on responses *after* cue onset but *before* stimulus onset. The responses when attention is directed into RF are compared to when attention is directed to a location outside the RF. Within cortex the coupling between alpha phase and gamma power increases during attention. There is alpha band (10–15Hz) coherence between the local field potentials (LFPs) in TEO and pulvinar, V4 and pulvinar as well as between TEO and V4. This alpha-band coherence also increased with attention. In addition, there was a small increase in gamma coherence, but only for the TEO-V4 pairs. The direction of interaction between brain regions was investigated using Granger causality. When attention was directed into the receptive fields, the alpha-band Granger causality was increased in the direction from the pulvinar to cortex, but between the two cortical areas it was unaltered.

The proposed interpretation of these results is that alpha oscillations in the pulvinar direct alpha oscillations in cortical areas that act to align gamma oscillations between areas, thereby improving information transmission [45]. Our model supports the potential role of alpha phase shifts in increasing efficiency of information transmission, reflected in enhanced gamma coherence and mutual information between firing rate fluctuations in the areas. As gamma coherence both depends on the degree of alpha alignment and whether sufficient activation is provided by bottom-up stimulus-related inputs or top-down inputs reflecting cognitive factors, the model would predict that when the stimuli are presented the intracortical gamma-band Granger causality would increase above its value that was measured during the cue period in [45].

Zhou and coworkers (experiment 2) use a similar but not identical task and measured from (ventral lateral) pulvinar, V4 and TE (a different area that is adjacent to TEO and has a different connectivity profile with the pulvinar [46]). In their task there is a cue in the center of the visual field that indicates the relevant location (target) in the stimulus array, which is comprised of different objects. The subject had to detect a small change in the target object and respond by making a saccade and the subject had to ignore changes in non-target objects. On some trials the cue preceded the onset of the stimulus array, thereby providing a measurement of how cortical networks re-organize to reflect the new focus of attention, prior to the onset of the stimulus response.

In Figure S4 of the work by Zhou and coworkers the Granger causality between LFP signals was determined in the period prior to stimulus onset. The gamma band GC from V4 to TE was enhanced by attention, and it was weak and not modulated by attention the other way around. The pulvinar-V4 GC was frequency selective, with the gamma band dominating from V4 to pulvinar, which was also strongly modulated with attention, and lower frequency band dominating in the pulvinar to V4 GC. In contrast to the study by Saalmann and coworkers, the alpha band GC from pulvinar to cortex was not modulated by attention. Interestingly our model does not require an increase in Granger causality (or coherence) in the alpha band from the pulvinar to the cortex, since it’s only the phase of cortical alpha oscillations that is modulated.

A possible interpretation of these results, which is consistent with our model, is that gamma band is representing stimulus-related information going from V4 to pulvinar, whereas the alpha band drive from pulvinar to V4 modulates the effectiveness of the V4-TE communication. Each band could reflect the activity of a different set of neurons, the balance between which varies with the particular group of neurons generating the LFP signal.

The differences in findings of experiment 1 and 2 can thus be caused both by differences in task setup as well as recording sites. The findings of either of the studies do not contradict the results of our model, though they raise several issues that could be investigated with additional model simulations.

### Structure and dynamics of pulvinar-cortical network

Our model is a severe simplification of the pulvinar-cortical network. Here we describe several aspects that are important for improving our model in future work.A key simplification of our model is representing the pulvinar activity simply as an alpha oscillation. It would be important to understand how this alpha oscillation could be generated by a network of spiking neurons. Our mechanism requires the generation of alpha oscillations with different phases depending on attentional demand. The existence of such a mechanism is supported by the finding of phase diverse thalamic projections in the cat thalamus. In the lateral geniculate nucleus of the thalamus, high threshold thalamic cortical (TC) neurons (HTC) are a subset of TC neurons that produce bursts activated by a cholinergic projection or by activation of metabotropic glutamate receptors [47–49]. This leads to the situation that when the alpha oscillation is generated by the HTC, the remaining TC can fire at different phases. The same area can thus project alpha with two different phases relative to the local alpha oscillation. In vitro studies, controlling the level of depolarization, shows that a finer grained control of the phase is also possible [47,48]. Under the assumption that neurons in the appropriate location of the primate pulvinar have similar properties, this extensive set of experiments support the notion that pulvinar excitatory drives can be tuned in terms of alpha phase, which is a necessity for the proposed mechanism. Therefore, extending the model with a physiologically realistic network model of the pulvinar alpha generator would be a great addition.

There is quite some evidence that the pulvinar-cortical interactions operate in both directions. The pulvinar is not only influencing cortex, but the cortex also projects back to the pulvinar [23]. Expanding our model by making the cortical areas influence the pulvinar would help to provide insight into the role of these feedback projections. Modeling stimulus representations in the pulvinar [24,50] and how bottom-up sensory information and top-down attentional control interact in the pulvinar could further increase our understanding of the pulvinar-cortical network.

Finally, an important aspect that we have not implemented in our model is the laminar organization of the cortex. Different cortical laminae support the processing of information from respectively cortical and subcortical sources. Feedforward projections from the pulvinar project to layer 4 of the cortex, while feedback projections from the pulvinar project to more superficial cortical layers [51–53]. This would cause the pulvinar to influence the higher and lower areas in the visual hierarchy in mechanistically different ways, relying on different cell types and circuitry. In addition, cortical oscillations have a laminar profile, in terms of the origin of alpha oscillations [54,55] relative to faster oscillations [56], which can differ going from earlier sensory areas (V1,A1, S1) to the ones further up in the hierarchy (V4, IT) [57]. Taking the laminar structure of the cortex into account is essential for making accurate predictions for electrophysiological measurements made with laminar electrical probes and should be considered in future work.

### Mechanism for cortical communication

Communication through coherence (CTC) was proposed as a principle to dynamically generate networks between cortical areas [2]. In its original form, it suggested that for areas to communicate effectively, they had to oscillate at the same frequency and the phase difference had to have a suitable value to align windows of excitability, for instance generated by a properly timed and synchronized inhibition [58]. The original principle of CTC did not come with a specific biophysical mechanism, but with predictions that were born out in experiment [59]. The lack of mechanism led to alternative interpretations [60,61] and modeling work arguing for it [62,63] and against it [64]. Experimentally, it was pointed out that gamma oscillations varied in frequency across time with a mean depending on the size and contrast of the stimulus [65]. This would make CTC difficult because the receiving area has to adjust to the ever-changing frequency. Nevertheless we now know that this is possible [66], presumably due to the entrainment properties of PING circuits. In a recent review paper [6], CTC has recently been revised to account for the new experimental results. In anesthetized monkeys, the relation between the probability of a spike in V1 eliciting a spike and V2 as a function of LFP phase was investigated [67]. CTC predicts that the spiking probability should be highest at the best local phase (i.e. in V2), but in experiment it was found to be at the best V1 phase, for which the V1 activity was the highest. Hence, the gamma was most effective at the time it corresponded to the highest number of active neurons. This is consistent with our model results (Fig 5). It supports a phase-based mechanism in the alpha band, which can influence information transmitted in the gamma band. Recently simulations have shown how a network with cross-frequency coupling can switch between a communication through coherence mechanism and a mechanism similar to the theta-gamma mechanism found in hippocampus [68]. This latter mechanism has been suggested to also play a role in visual processing [69]. Further investigations need to be conducted to find out how alpha phase changes would influence this mechanism.

### Experimental predictions

Our model is consistent with several recent experimental findings. To further validate our model we here formulate explicit predictions that can be tested by re-analyzing existing electrophysiological data or by conducting additional experiments. A clear prediction from our model is that the alpha phase difference between two connected cortical areas should be correlated with the gamma coherence between both areas. Such experiments can be conducted in animals using intracranial field recordings or in humans using MEG. A paradigm can be used in which the need for communication between the regions is manipulated; e.g. an attention type of paradigm. The alpha phase difference between two cortical areas should be different when comparing the attention into the receptive field condition to that with attention directed outside the receptive field condition. On a trial by trial basis, the difference in alpha phase should be correlated with gamma coherence, as well as task performance.

Finally, methods such as DREADD [70] and optogenetics [71] can be used to actively manipulate the phase of cortical alpha oscillations in animal studies. These techniques are well suited to target very specific groups of neurons in the cortex or pulvinar. By active individual manipulation of the alpha phase in two connected cortical areas we predict an influence on gamma coherence between these areas. These techniques are currently well-established for use in rodents, but are in the process of being adapted for primates use [72].

### Conclusions

We have shown a possible role for the alpha rhythm in coordinating cortical communication. By controlling the alpha phase difference between cortical areas we were able to influence the effectiveness of communication and the processing of stimuli. In bidirectionally coupled networks the alpha phase difference determined the direction of communication. The pulvinar would be an ideal candidate for controlling this mechanism. We formulated a number of experimental tests to support the hypothesized mechanisms and further clarify the role of the pulvinar in the process of selective attention.

## Models & methods

### Model overview

To study communication between neuronal populations in different cortical areas we modeled each population as a local network of strongly interconnected neurons (Fig 2). Each population consisted of 400 regular spiking (RS) excitatory pyramidal neurons, 75 fast spiking (FS) interneurons and 25 low threshold spiking (LTS) interneurons [73]. Connectivity within each population was all-to-all: each neuron projected to all other neurons, representing the strong connectivity of a local cortical population. In most simulations, two of these populations were connected, each population representing a part of a different cortical area within the visual cortex. Depending on the simulation experiment, the populations were connected either uni-directionally or bi-directionally. Each neuron in each population received an independent random noise current inputs that was uncorrelated between neurons. Unless stated otherwise, the input was modulated with a 10 Hz (alpha frequency) sinusoidal oscillation, simulating the coordinated input activity from the pulvinar.

#### Neuron model

To simulate the potential dynamics of the different neuron types we used the model proposed by Izhikevich [31]. This simple model is represented by the following equations

$$\dot{v}=0.04v^{2}+5v+140-u+I$$

$$\dot{u}=a(bv-u)$$

together with a reset condition: when v crosses 30 mV from below: an action potential is called and the variables are reset according to

v←c,

u←u+d

Here *v* represents the membrane potential of the simulated neuron, *I* controls the amount of current flowing into the neuron (level of depolarization) and *u* is a slow 'recovery' variable which can be interpreted as the action-potential induced activation of K^+^ or inactivation of Na^+^ currents. The four dimensionless parameters *a*, *b*, *c* and *d* determine the dynamical characteristics of the neuron, the additionally numerical constants set spike threshold and rest potential. The parameter values are given in Table 1for each of the different neuron types used in the simulation.

**Table 1:** Parameter values for the different neuron types used in the model.

Parameter	RS	FS	LTS
**a**	0.02	0.1	0.02
**b**	0.2	0.2	0.25
**c**	-65	-65	-65
**d**	8	2	2

RS, Regular spiking; FS, Fast spiking; LTS, Low-threshold spiking.

In this version of the Izhikevich model only v has physical units, i.e. mV. The other variables are dimensionless. To compare with physiologically realistic values for the current we converted the dimensionless I parameter to units of pA. We did this by comparing the firing rate versus input current (FI) curve of our model to that of a model with input current expressed in pA units (S7 Fig)[74].

#### Connectivity

For each pair, the connection strength between neurons was randomly drawn from a uniform distribution, whose properties were determined by the both the type of the sending and receiving neuron. Table 2 gives an overview of the average connection strengths between each neuron type, based on experimental findings [75].

**Table 2:** Connectivity strength between the different neuron types.

Neuron type	RS	FS	LTS
**RS**	0.0375	-0.25	-0.3
**FS**	0.125	-0.15	-0.1
**LTS**	0.125	-0.1	0

RS, Regular spiking; FS, Fast spiking; LTS, Low-threshold spiking. Values represent the range of a uniform distribution (starting at 0 ending at the value listed in the table) from which connection strengths between neurons were drawn randomly.

These connection strengths represent the amount of current that enters the receiving neuron after a spike of the sending neuron. These evoked post-synaptic currents decreased exponentially after firing with decay constants τ_E_ = 2.5 ms for excitatory currents and τ_I_ = 6 ms for inhibitory currents, based on the typical decay values for AMPA and GABA receptors, respectively, reported in Ref. [76].

#### Input

The input current to each neuron consisted of evoked post-synaptic currents caused by other neurons, plus random noise

$$I_{network,n}=\sum_{i\neq n}E_{i}+\varepsilon_{n}$$

Where *I*_*network*,*n*_ is the input current to neuron *n*, *E*_*i*_ is the evoked post-synaptic current caused by neuron *i* and *ε*_*n*_ is the random noise input drawn independently for each neuron and on each time step from a normal distribution *N(μ*,*σ)* effectively generating white noise. Each of the three different neuron types had a separate noise distribution of which the parameters were adjusted to produce a realistic spiking behaviour (Table 3).

**Table 3:** Noise parameters for different neuron types.

Parameter	RS	FS	LTS
**μ**	4	5	4
**σ**	6	4	4

μ, mean of normal distribution generating the noise; σ, standard deviation of normal distribution generating the noise.

Besides this input from neurons within the network, neurons also received a modulatory sinusoidal input of 10 Hz

$$I_{mod,n}=A\;sin(\varphi (t))$$

with *A* the amplitude of the modulation and *φ(t)* the phase of the modulation as a function of time. The amplitude used in all experiments was 23 pA unless stated otherwise.

Most studies indicate that alpha oscillations in the visual cortex are of an inhibitory nature [8]. Projections from the thalamus to the cortex also have more synapses on inhibitory neurons and cause stronger evoked postsynaptic potentials in inhibitory cells [77]. For this reason only inhibitory neurons in the model received the alpha modulation. The alpha rhythm was transferred to the excitatory neurons by way of the strong interneuron to excitatory cell projection. Our initial simulations showed there was no qualitative difference between modulating the inhibitory neurons or the excitatory neurons, except that inhibitory neurons needed stronger modulation, because they are fewer in number.

The inhibitory neurons in each population received alpha currents with the same modulation amplitude and phase, but between the two populations there was a constant phase-difference. The phase of the modulation, *φ (t)*, was created according to the Kuramoto model [78]. There were frequency fluctuations around an average frequency of 10 Hz, while the phase coherence between both populations was maintained through a coupling with strength J:

$${\dot{\varphi }}_{1}(t)=\omega_{0}+\eta_{1}(t)+J\;\sin\left(\varphi_{2}(t)-\varphi_{1}(t)\right)$$

$${\dot{\varphi }}_{2}(t)=\omega_{0}+\eta_{2}(t)+J\;sin(\varphi_{1}(t)-\varphi_{2}(t))$$

with *φ*_*1*_*(t)* and *φ*_*2*_*(t)* being the modulation phases of population 1 and 2 respectively, *ω*_*0*_ the average frequency of 2π ⋅ 10 Hz, *η(t)* a noise parameter and *J* a coupling strength parameter that determines how well the constant phase offset is maintained. We used a value of 0.2 for J, such that the phase difference between both populations was well maintained. For convenience we will refer to the phase relationship between population 1 and 2 using the relative phase-difference, i.e.

$$\varphi_{1}(t)-\varphi_{2}(t)=\Delta \varphi$$

S8 Fig gives an example of the waveforms and power spectrum of the alpha modulation.

### Simulations and analyses

In order to investigate cortical communication the following analyses were performed. All simulations and analyses were performed in MATLAB R2012b.

Our neuron model was numerically integrated using an adaptation of Euler’s method [31]. Every simulation step consisted of 2 sequential steps of 0.5 ms for numerical stability. After each simulation step spikes were detected. Specifically, we defined spike times as the time at which the spike reached its peak potential of 30 mV. The mean firing rate was determined separately for each neuron type as the number of firings of a neuron divided by the trial time, averaged over all neurons of the same type

$$r=\frac{1}{NT}\sum_{i}n_{i}$$

with *r* being the firing rate, *N* the number of neurons of a certain type, *T* the trial duration and *n*_*i*_ the number of firings of neuron *i*. We defined *r*_*RS*_, *r*_*FS*_ and *r*_*LTS*_ to be the firing rates for the regular spiking, fast spiking and low threshold spiking neurons, respectively.

Many of the analyses were performed on the spike timing histograms (STHs) for each neuron type seperately. The STHs were defined as the total number of firings of a neuron type within a certain time bin

$$STH=\sum_{i}X_{i}(t)$$

Where

$$X_{i}(t)=\left\{ \begin{array}{l} 1,\;\mathrm{if}\;f_{j}^{i}\in (t,t+\Delta t)\\ 0\;\mathrm{otherwise} \end{array}\right.$$

with *X*_*i*_*(t)* determining whether neuron *i* fired within the time bin at time *t*, *f*^*i*^_*j*_ the time of the *j*th spike of neuron *i*. The time bin had a width of *Δt = 1ms*.

#### Power

In order to investigate the behaviour of oscillations at different frequencies in the simulations we used power spectrum analysis. This analysis was done using the CHRONUX toolbox [79]. This toolbox performs a multi tapered fast discrete Fourier transform on the data. The elements of the Fourier transform are given by

$$X(f)=\sum_{j=1}^{N}x(j)e^{-\frac{2\pi (j-1)(f-1)}{N}}$$

with *x(j)* being the *j*th data point, *N* the number of data points and *f* is the frequency. Before performing the fast Fourier transform the data *x(j)* was convolved with multiple tapers, the number of which depended on the spectral resolution, *W*, needed and the time window, *T*, across which the tapers were calculated, jointly expressed as the time-half-bandwidth product, NW. Unless stated otherwise the power was measured over a duration of T = 2000 ms with a time-half-bandwidth product, *NW = 5* and a resulting spectral resolution *W = 2*.*5* Hz. To obtain the power spectral density, the absolute values of the (multi-tapered) Fourier elements were taken

$$S(f)={\left|X(f)\right|}^{2}$$

#### Coherence

To quantify communication between both neuronal populations, we analysed the coherence between temporal dynamics of both populations. The CHRONUX toolbox was used to calculate the coherence. The coherence is based on the cross-spectral density between two signals

$$C_{xy}(f)=\frac{{\left|S_{xy}(f)\right|}^{2}}{S_{xx}(f)S_{yy}(f)}$$

where *S*_*xy*_*(f)* is the cross spectral density and *S*_*xx*_*(f)* and *S*_*yy*_*(f)* are the respective autospectral densities. Tapers were applied the same way as for the power spectrum. Unless stated otherwise the coherence was measured over a duration of T = 2000 ms with a time-half-bandwidth product *NW = 5* and spectral resolution *W = 2*.*5Hz*.

#### Granger causality

During some of our simulations we studied a network with bi-directional connectivity between both neural populations. For these simulations we determined the directionality of communication using Granger causality, which was calculated using the Multivariate Granger Causality (MVGC) MATLAB toolbox [80].

The Granger causality from an area A to an area B determines how much better the activity in area B can be explained by including the past activity of area A in addition to the past activity of area B. The analysis performed by the MVGC toolbox is based on a vector autoregressive model (VAR) of the multivariate time-series ***U*** which makes a model of the time-series ***U***_*t*_ at time t given the history of this time-series, ***U***_*t-k*_, with lags k taken up to order *p*

$$U_{t}=\sum_{k=1}^{p}A_{k}U_{t-k}+\varepsilon_{t}$$

where A_k_ are the regression coefficients and ***ε***_*t*_ is the residual noise, which is turned into a residual noise covariance matrix

$$\Sigma =Cov(\varepsilon_{t})$$

Because we would like to know the frequency-resolved Granger causality, we used the spectral Granger causality. For this measure the stochastic process ***U***_*t*_ is converted to an auto-covariance sequence *Γ*_*k*_

$$\Gamma_{k}=Cov(U_{t},U_{t-k})$$

From this we can obtain the cross power spectral density (CPSD) via a Fourier transform

$$S(\lambda )=\sum_{k=-\infty }^{\infty }\Gamma_{k}e^{-ik\lambda }\;0\leq \lambda \leq 2\pi$$

For a VAR process this CPSD is given as

$$S(\lambda )=H(\lambda )\Sigma H{\left(\lambda \right)}^{*}$$

where *H(λ)* is a transfer function that is defined as the inverse matrix of the Fourier transform of the regression coefficients

$$H(\lambda )={\left(I-\sum_{k=1}^{p}A_{k}e^{-ik\lambda }\right)}^{-1}$$

and the * denotes the complex conjugate and transpose.

In case we want to know the Granger causal influence of a time-series *Y* on another time-series *X* we have

Ut=(XtYt)

and we can write the CPSD as

$$S(\lambda )=\left(\begin{array}{cc} S_{xx}(\lambda ) & S_{xy}(\lambda )\\ S_{yx}(\lambda ) & S_{yy}(\lambda ) \end{array}\right)$$

with individual elements given as

$$S_{xx}(\lambda )=H_{xx}(\lambda )\Sigma_{xx}H_{xx}{\left(\lambda \right)}^{*}+2R\{H_{xx}(\lambda )\Sigma_{xy}H_{xy}{\left(\lambda \right)}^{*}\}+H_{xy}(\lambda )\Sigma_{yy}H_{xy}{\left(\lambda \right)}^{*}$$

where R denotes the real part and ‘*’ denotes the Hermitian conjugate, which involves taking the matrix transpose and the complex conjugate of each element. This expression can always be converted to a form where

$$\Sigma_{xy}=0$$

leaving the Granger causality invariant [81]. In this case we get the expression

$$S_{xx}(\lambda )=H_{xx}(\lambda )\Sigma_{xx}H_{xx}{\left(\lambda \right)}^{*}+H_{xy}(\lambda )\Sigma_{yy}H_{xy}{\left(\lambda \right)}^{*}$$

where the first part is an intrinsic term, representing the contribution of the local process and the last part a causal term, representing the contribution from the external process.

The spectral Granger causality is then given as the total spectral density divided by the part caused by the intrinsic term

$$f_{Y\to X}(\lambda )=\ln\frac{\left|S_{xx}(\lambda )\right|}{\left|S_{xx}(\lambda )-H_{xy}(\lambda )\Sigma_{yy}H_{xy}{\left(\lambda \right)}^{*}\right|}$$

which thus gives a measure for the causal strength of the external process in comparison to the intrinsic process.

The conditional case is an extension of this and we refer to [80] for a detailed explanation of how the MVGC toolbox implements this.

#### Support vector machines

The support vector machines used for classifying the different stimuli define the hyperplane that optimally separates the two classes of stimuli. They do this by maximizing the margin, that is the maximum distance between two hyperplanes parallel to the separating hyperplane that do not contain any interior data points. If we have data vectors *x*_*j*_ that belong to classes *y*_*j*_ *= ±1*, we can define a hyperplane as

f(x)=x∙β+b

where *β* has the same dimension as *x* and *b* is a scalar. The problem can then be defined as finding the *β* and *b* that minimize *|| β||* in such a way that for all data points

$$y_{j}f(x_{j})\geq 1$$

This problem can be solved using a quadratic programming algorithm; in our case the standard function ‘svmtrain’ in MATLAB.

The classification of a vector *z* can be done using the optimal solution (β*,b*)

$$class(z)=sign(z∙\beta^{*}+b^{*})$$

#### Mutual information

To quantify information transfer in our model we used the mutual information measure. The mutual information of our system quantifies how well stimulus information can be extracted from responses of the system. It uses the entropy of the responses r

$$H(r)=-\sum_{r}p(r)\;\log\;p(r)$$

which measures the variability of responses in the system under influence of the different stimuli. Here the response r is the number of spikes in a period of 30ms after stimulus onset. Not all of this variability is caused by the stimulus though, as some of it is due to the intrinsic properties of the system. To compensate for this the marginal entropy

$$H(r|s)=-\sum_{s}p(s)\sum_{r}p(r|s)\;\log\;p(r|s)$$

which gives the variability of responses under influence of the same stimulus s, is subtracted. The mutual information between response r and stimulus s is thus given by

I(r,s)=H(r)−H(r|s)

In terms of probability distributions this gives

$$I(r,s)=\sum_{s}p(s)\sum_{r}p(r|s)\;\log\frac{p(r|s)}{p(r)}$$

which is always positive. The maximum mutual information depends on the amount of stimuli encoded. We used a logarithmic base of 2 so that mutual information was given in bits. For a system with 2 stimuli as used in our simulations the maximal mutual information is then 1 bit.

#### Wavelet analysis

Some of our analyses required determining the phase of the different oscillatory frequencies at a high temporal resolution. To obtain this temporal progression of phase we used a wavelet analysis. Complex Morlet wavelets were convolved with the signal to obtain a complex number describing the power and phase for every frequency and time-step. The complex Morlet wavelet is described by

$$\psi (t,f)=Ae^{-\frac{t^{2}}{2\sigma_{t}^{2}}}e^{2i\pi f_{0}t}$$

with *f*_*0*_ the center frequency of the wavelet and *σ*_*t*_ the bandwidth parameter of the gaussian envelope. The bandwidth of the complex wavelet scaled with 1/f_0_ such that at every frequency the envelop of the wavelet was equal to one period of the centre frequency and the support of the wavelet was infinite. The complex wavelet transform is found by convolving the signal, *x(t)*, with this wavelet

$$CWT_{x}(t,f)=\sqrt{\frac{f}{f_{0}}}\int_{-\infty }^{\infty }x(\tau )\psi^{*}\left(\frac{f(\tau -t)}{f_{0}}\right)d\tau$$

where ψ^*^ is the complex conjugate of the Morlet wavelet. The power and phase of the signal at a particular frequency can be calculated in a time-resolved fashion using the complex wavelet transform:

$$P_{x}(t,f)={\left|CWT_{x}(t,f)\right|}^{2}$$

$$\phi_{x}(t,f)=arg(CWT_{x}(t,f))$$

The main advantage of wavelet analysis over traditional short-time Fourier analysis is that it has better temporal resolution for higher frequencies, enabling better tracking of the phase at different time points.

## Supporting information

S1 Fig Effect of alpha phase difference when the alpha rhythm modulates the input to the excitatory neurons directly. Gamma coherence between area 1 and area 2 when the alpha rhythm modulates input to the excitatory neurons (A). The phase (B) and amplitude (C) coherence both depend on the alpha phase difference, but the phase coherence is more strongly modulated. The amplitude coherence is strongly biased due to the similar alpha modulation in both areas. When comparing the coherence spectrum for the optimal alpha phase (-90°, red) with the least optimal alpha phase (90°, blue), we can see that the effect on coherence is limited to the gamma band (D).
(TIF)

S2 Fig Rastergrams for different alpha modulation amplitudes. Rastergram of neuronal firing when there is no alpha modulation (A), intermediate alpha modulation of 23 pA (B) or maximal alpha modulation of 45 pA (C). Only under the highest modulation strength the activity in the troughs of the alpha oscillation is almost fully silenced. The more moderate amplitude of (B) was used for all further analysis though.
(TIF)

S3 Fig Effects of alpha phase difference when alpha rhythm is modelled as Sin function instead of Sin instead of Sin^2^. Firing rates for the excitatory population (red) and inhibitory population (blue) of area 1 (A) and area 2 (B) under a sinusoid modulation. The difference between the gamma coherence for the optimal alpha phase difference (red) and that for the least optimal alpha phase difference (blue) increases with higher alpha amplitude (C).
(TIF)

S4 Fig Average effect of alpha phase difference on stimulus response. (A) Averaged across all stimulus onset phases in area 1 we find a clear dependence of the stimulus response on the alpha phase difference between both areas.
(TIF)

S5 Fig Effect of alpha phase difference on information transmission. (A) Classification error depends on alpha phase difference. (B) Averaged over all phases for stimulus onsets in area 1 we find a modulation of the mutual information similar to that shown in Fig 4A.
(TIF)

S6 Fig The directionality of the interaction between two areas fluctuated when the alpha phase difference is zero. (A) When the alpha phase difference between both areas is zero the directions alternate such that there is always causality in just 1 direction.
(TIF)

S7 Fig Firing rates versus input current for different neuron types. (A) Regular spiking, (B) fast spiking and (C) low threshold spiking.
(TIF)

S8 Fig Examples of modulatory alpha oscillations. To give an idea about the fluctuation of the alpha oscillation compare the green line in (A) representing an oscillation without fluctuations to the blue line in (A) where the alpha oscillation fluctuated. The power spectral density plot of the resulting fluctuating oscillation can be found in (B).
(TIF)
