# Supplementary figures and images for: Top-down control of cortical gamma-band communication via pulvinar induced phase shifts in the alpha rhythm
Source: PLoS Comput Biol. 2017 May 4;13(5):e1005519. doi: 10.1371/journal.pcbi.1005519 (PMC5436894; doi:10.1371/journal.pcbi.1005519)

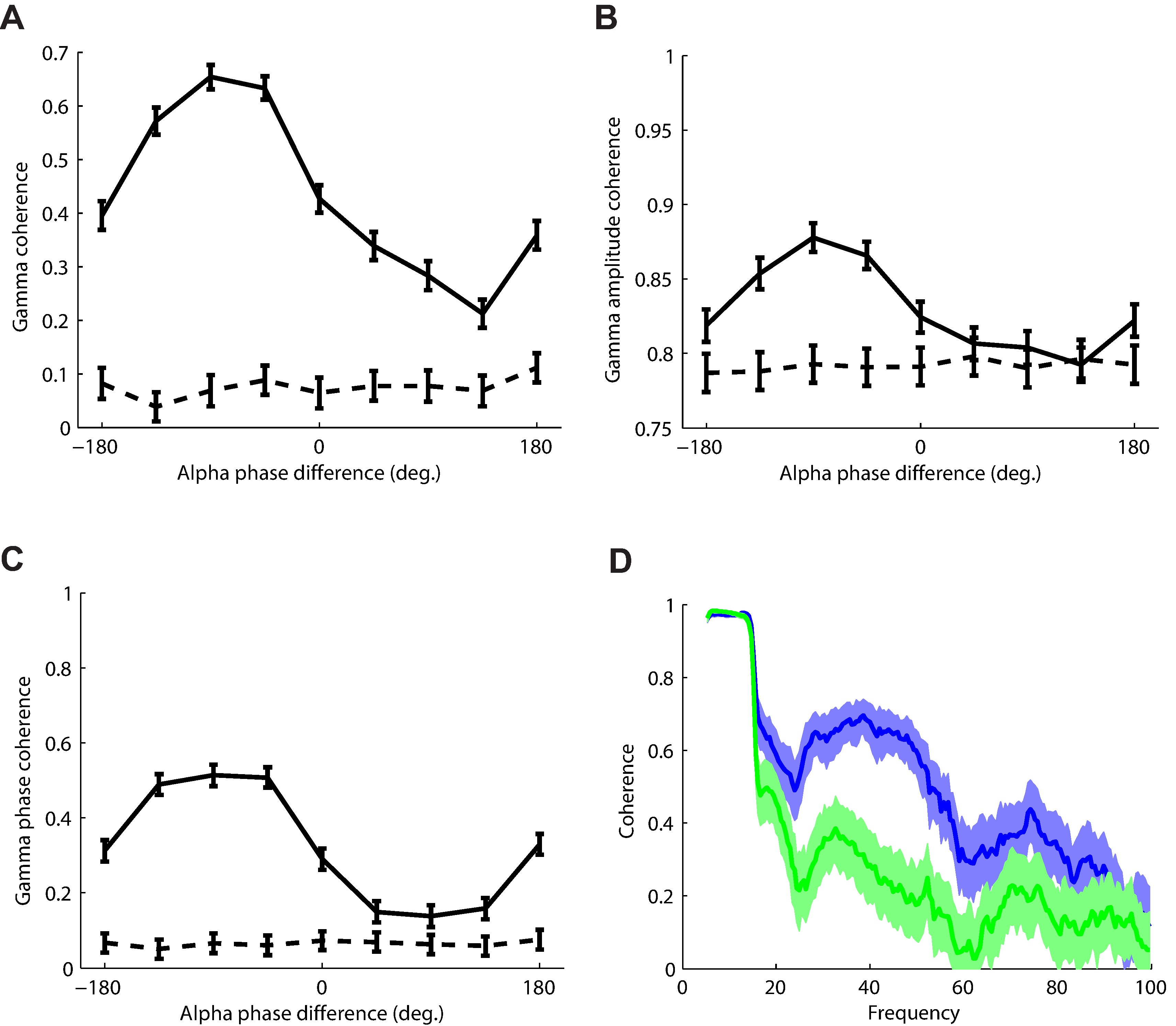

Supplement: S1 Fig — Gamma coherence between area 1 and area 2 when the alpha rhythm modulates input to the excitatory neurons (A). The phase (B) and amplitude (C) coherence both depend on the alpha phase difference, but the phase coherence is more strongly modulated. The amplitude coherence is strongly biased due to the similar alpha modulation in both areas. When comparing the coherence spectrum for the optimal alpha phase (-90°, red) with the least optimal alpha phase (90°, blue), we can see that the effect on coherence is limited to the gamma band (D). (TIF) [file pcbi.1005519.s001.tif]

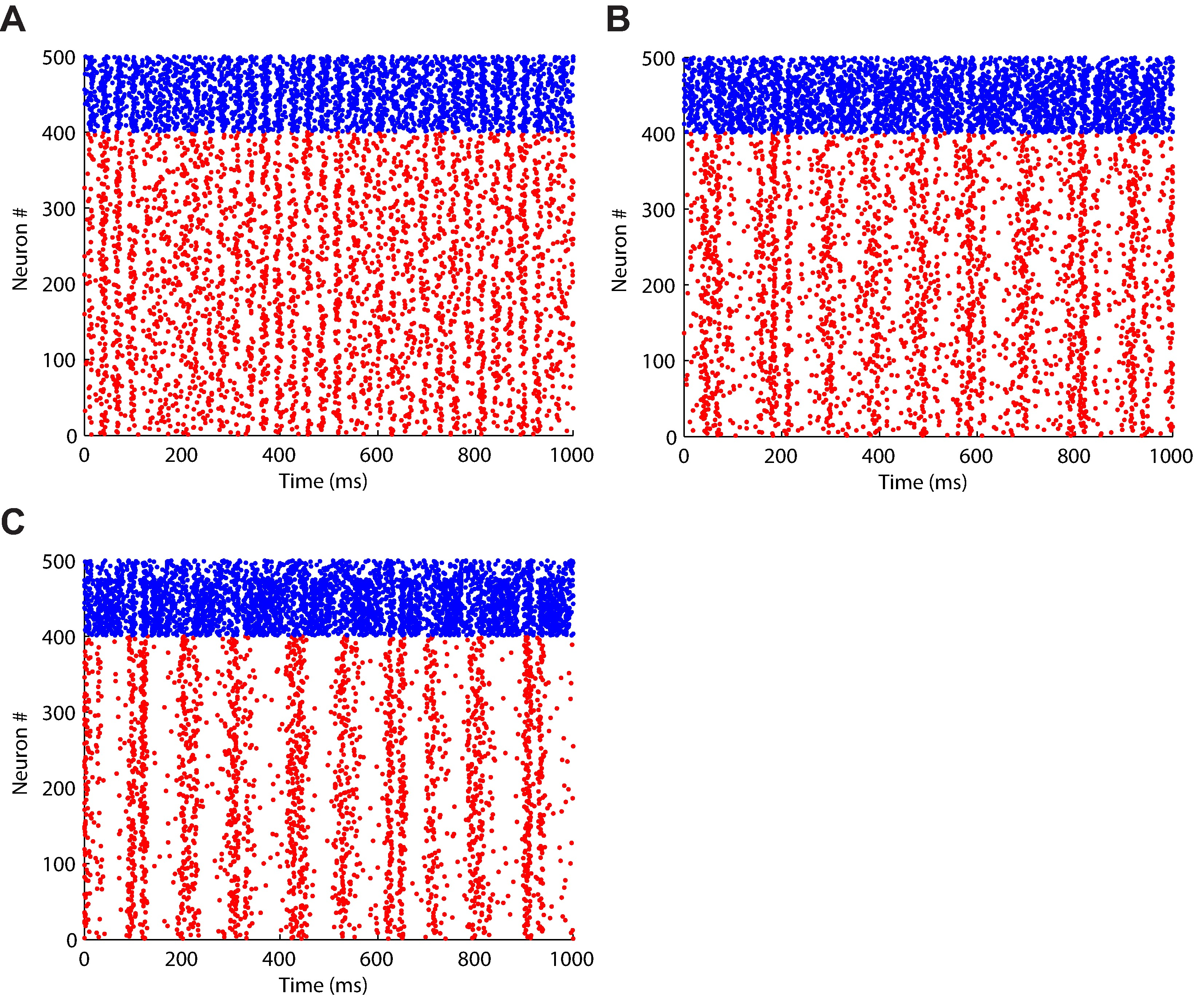

Supplement: S2 Fig — Rastergram of neuronal firing when there is no alpha modulation (A), intermediate alpha modulation of 23 pA (B) or maximal alpha modulation of 45 pA (C). Only under the highest modulation strength the activity in the troughs of the alpha oscillation is almost fully silenced. The more moderate amplitude of (B) was used for all further analysis though. (TIF) [file pcbi.1005519.s002.tif]

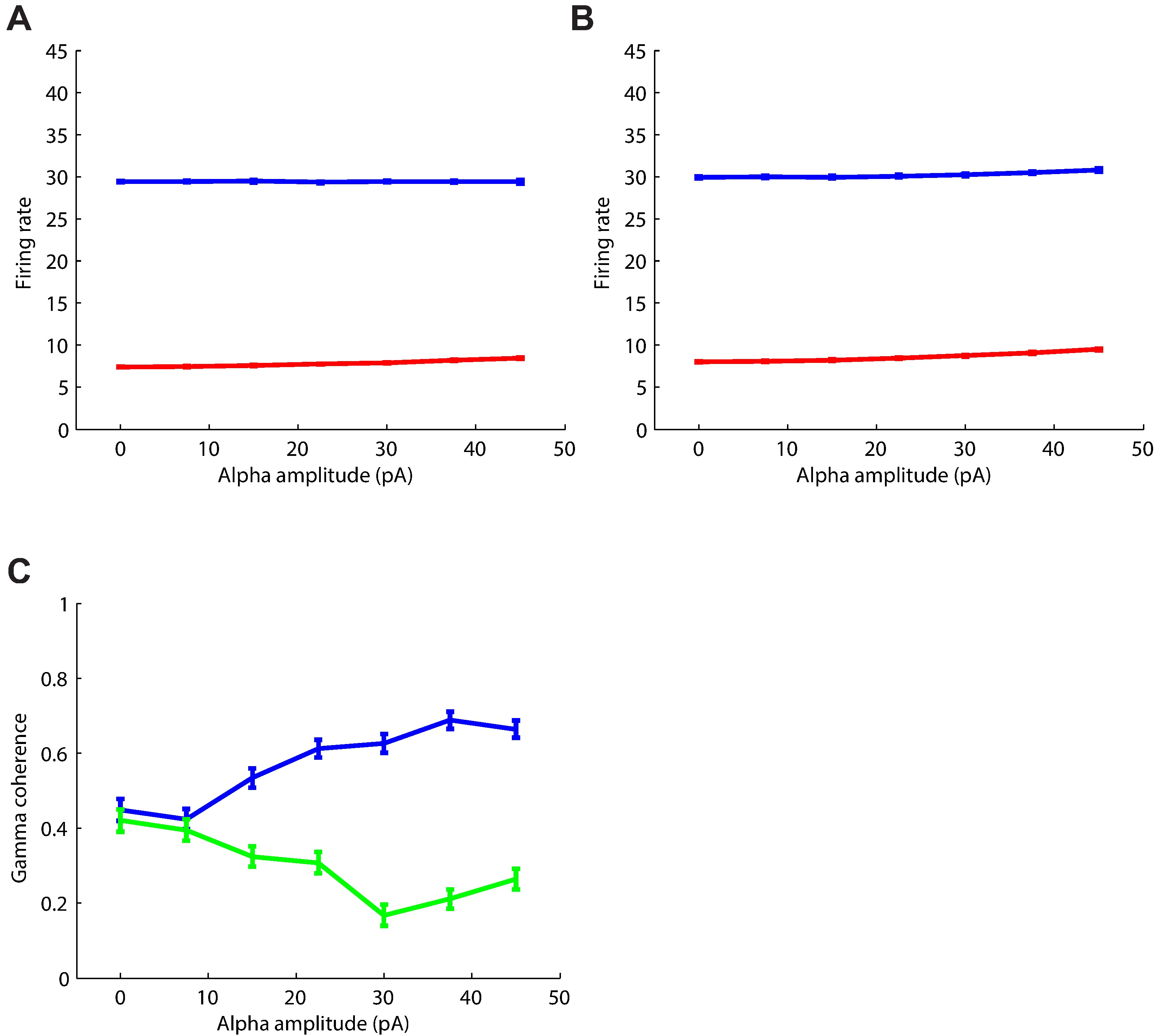

Supplement: S3 Fig — Firing rates for the excitatory population (red) and inhibitory population (blue) of area 1 (A) and area 2 (B) under a sinusoid modulation. The difference between the gamma coherence for the optimal alpha phase difference (red) and that for the least optimal alpha phase difference (blue) increases with higher alpha amplitude (C). (TIF) [file pcbi.1005519.s003.tif]

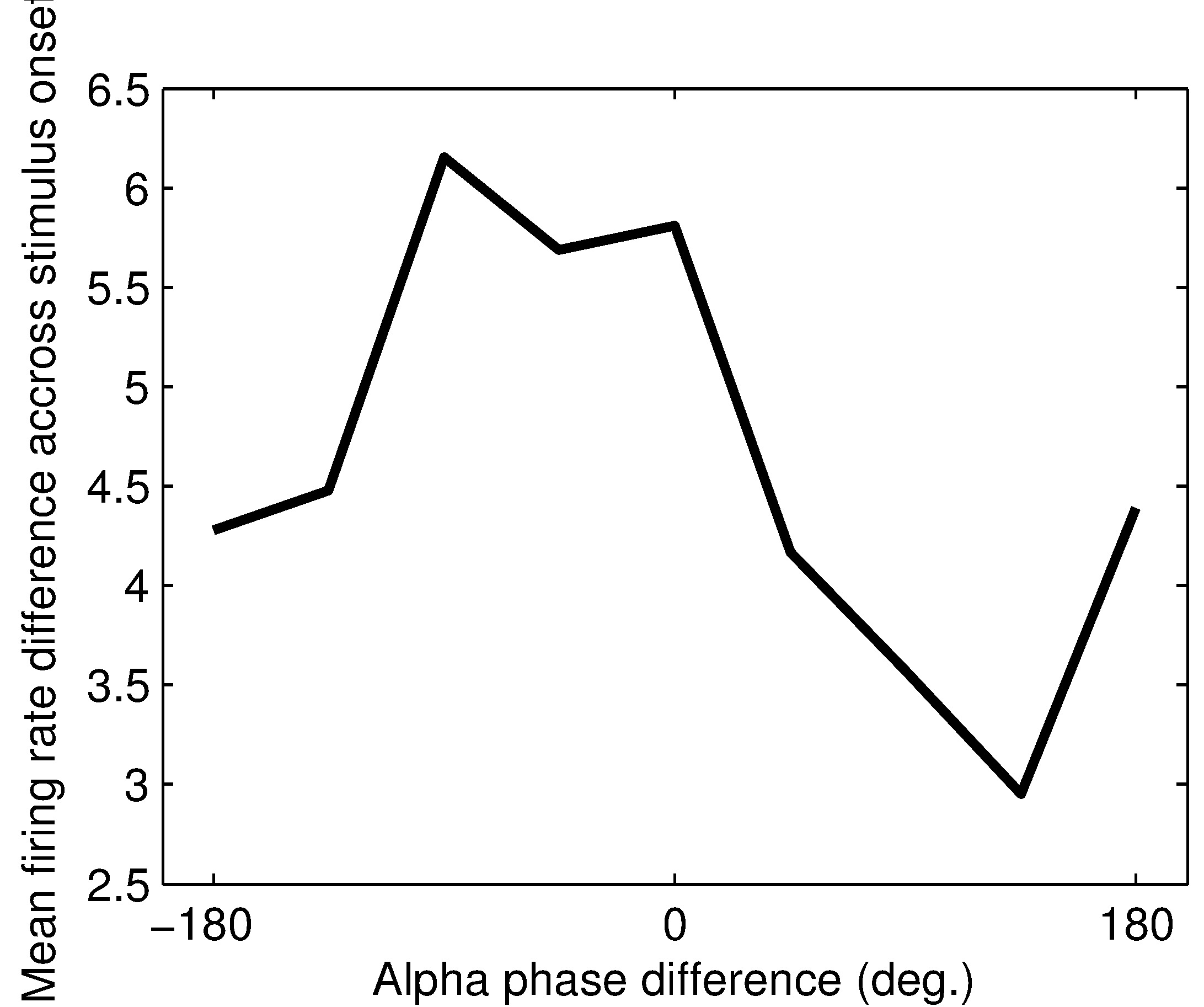

Supplement: S4 Fig — (A) Averaged across all stimulus onset phases in area 1 we find a clear dependence of the stimulus response on the alpha phase difference between both areas. (TIF) [file pcbi.1005519.s004.tif]

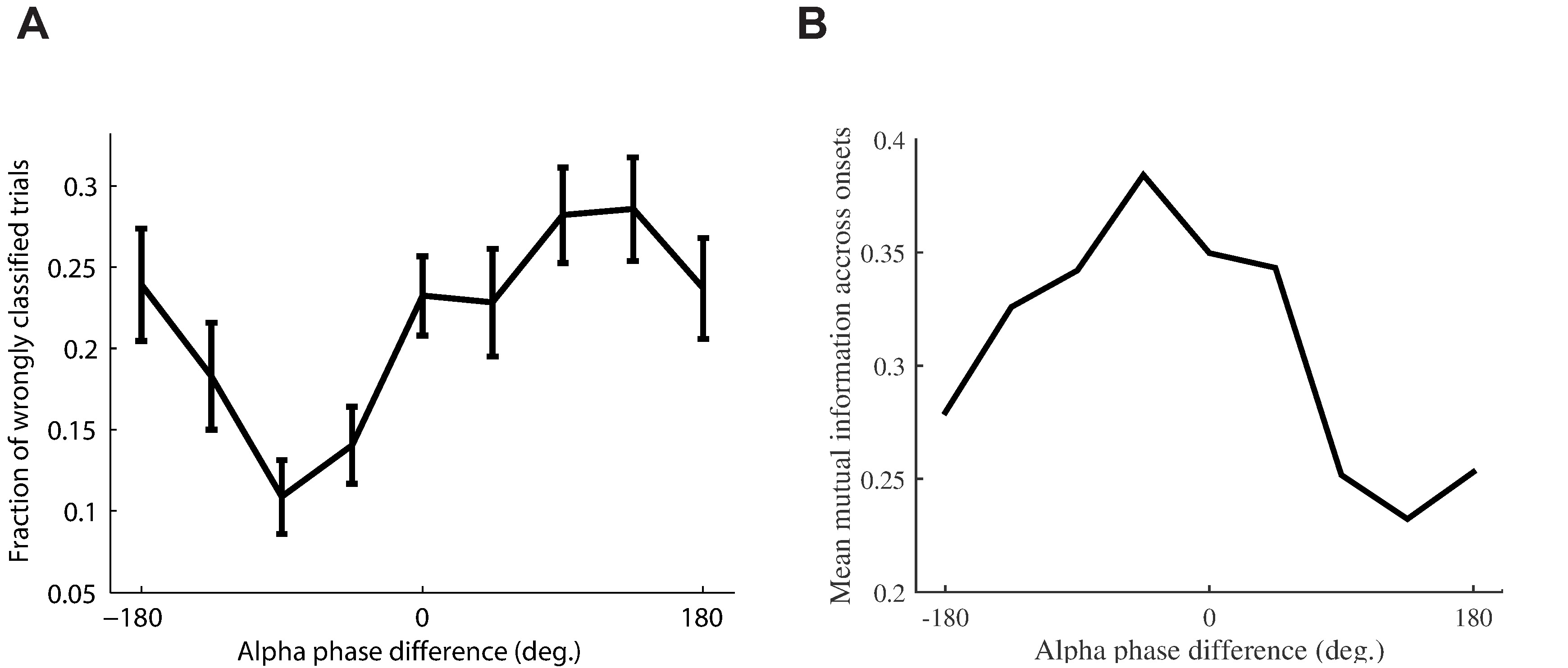

Supplement: S5 Fig — (A) Classification error depends on alpha phase difference. (B) Averaged over all phases for stimulus onsets in area 1 we find a modulation of the mutual information similar to that shown in Fig 4A. (TIF) [file pcbi.1005519.s005.tif]

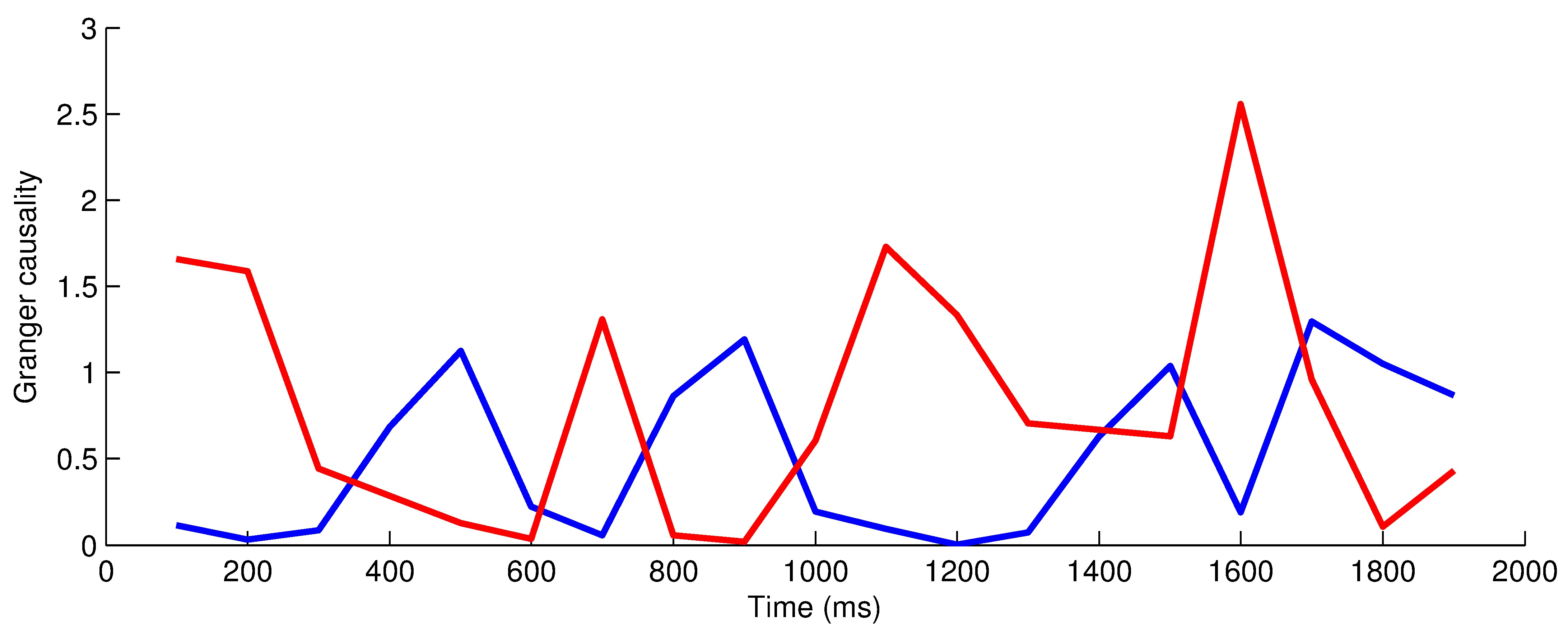

Supplement: S6 Fig — (A) When the alpha phase difference between both areas is zero the directions alternate such that there is always causality in just 1 direction. (TIF) [file pcbi.1005519.s006.tif]

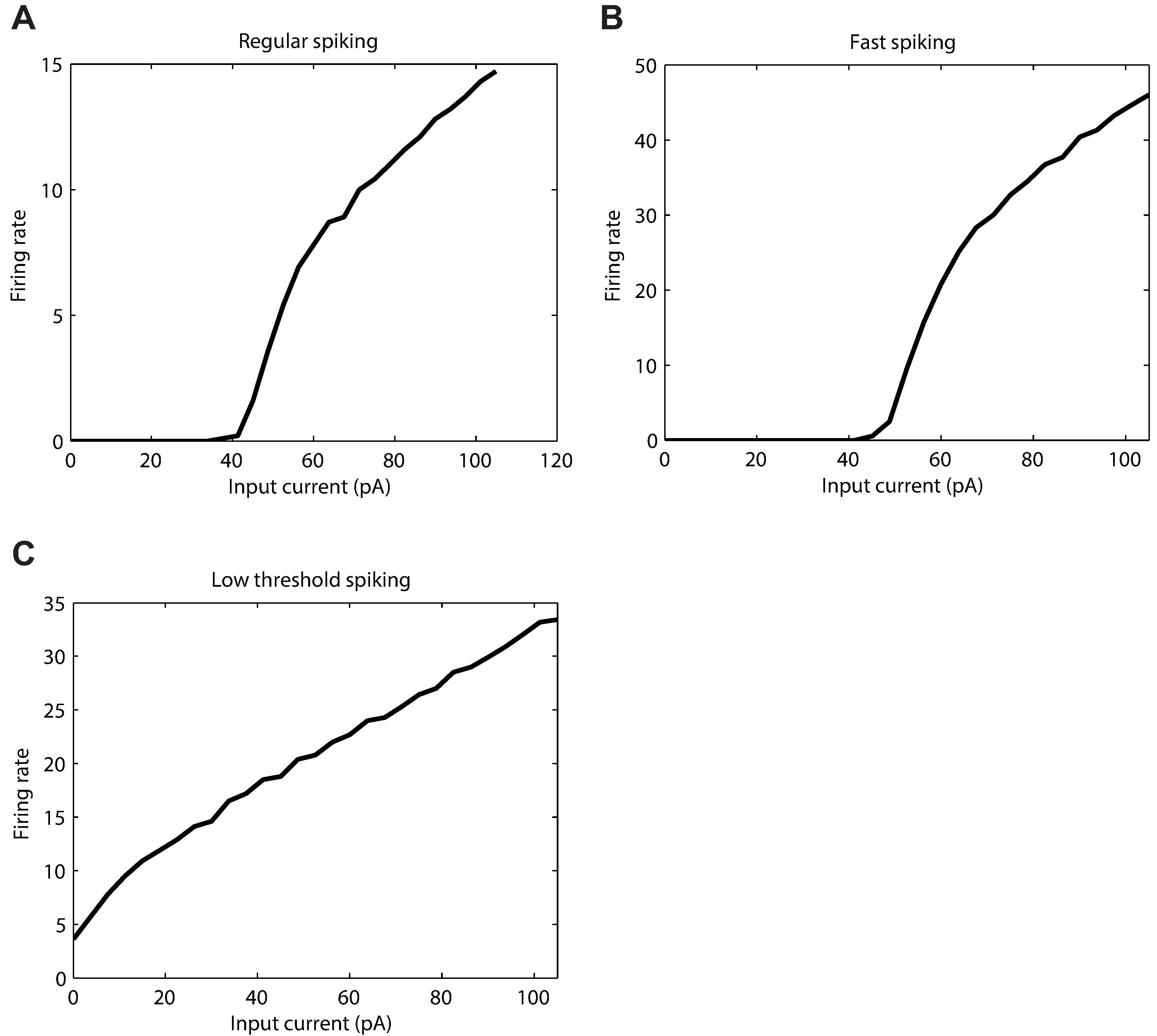

Supplement: S7 Fig — (A) Regular spiking, (B) fast spiking and (C) low threshold spiking. (TIF) [file pcbi.1005519.s007.tif]

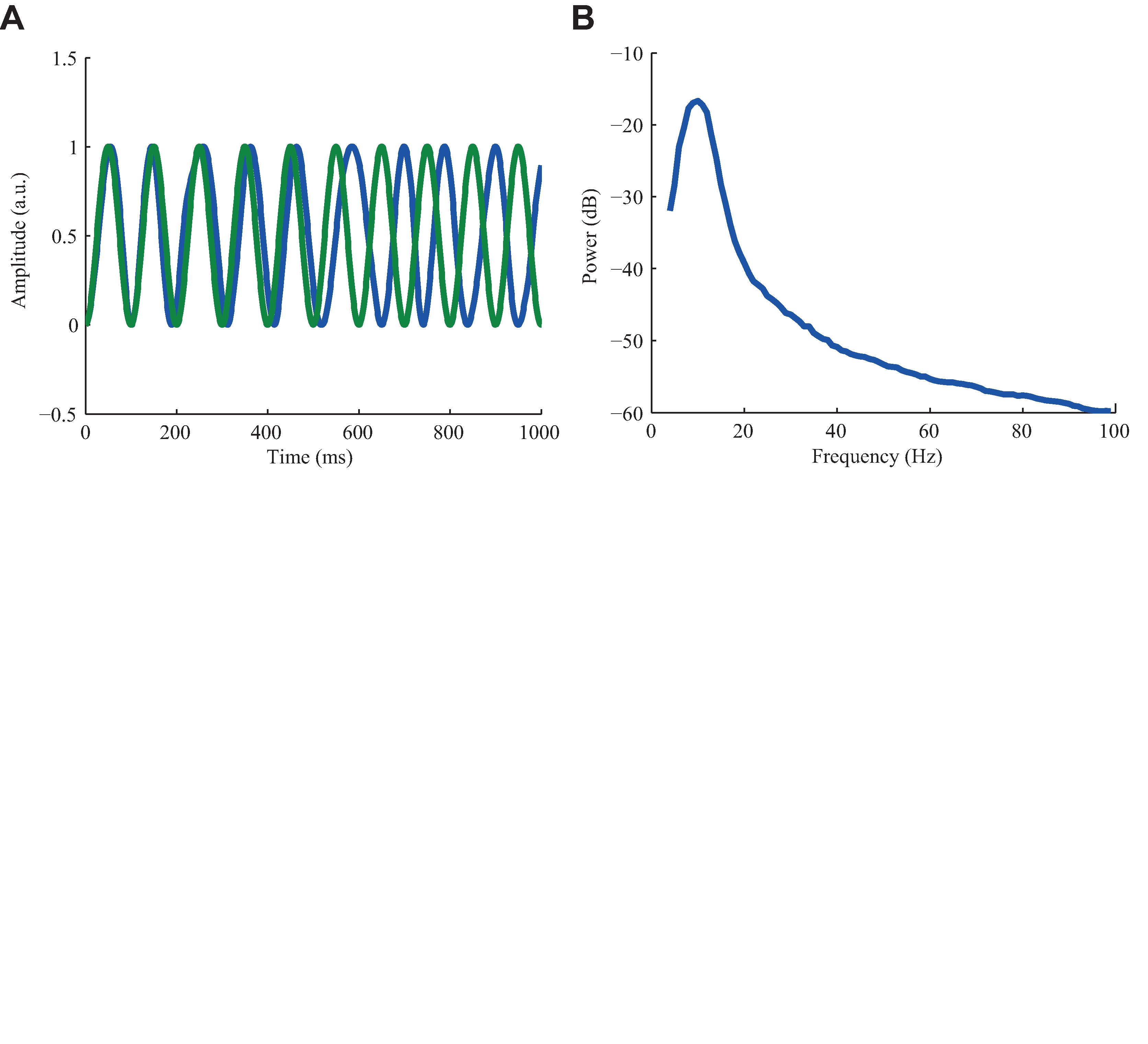

Supplement: S8 Fig — To give an idea about the fluctuation of the alpha oscillation compare the green line in (A) representing an oscillation without fluctuations to the blue line in (A) where the alpha oscillation fluctuated. The power spectral density plot of the resulting fluctuating oscillation can be found in (B). (TIF) [file pcbi.1005519.s008.tif]
